# Scaffold RNA engineering in type V CRISPR-Cas systems: a potent way to enhance gene expression in the yeast *Saccharomyces cerevisiae*

**DOI:** 10.1093/nar/gkad1216

**Published:** 2023-12-24

**Authors:** Lifang Yu, Mario Andrea Marchisio

**Affiliations:** School of Pharmaceutical Science and Technology, Tianjin University, 92 Weijin Road, 300072 Tianjin, China; School of Pharmaceutical Science and Technology, Tianjin University, 92 Weijin Road, 300072 Tianjin, China

## Abstract

New, orthogonal transcription factors in eukaryotic cells have been realized by engineering nuclease-deficient CRISPR-associated proteins and/or their guide RNAs. In this work, we present a new kind of orthogonal transcriptional activators, in *Saccharomyces cerevisiae*, made by turning type V CRISPR RNA into a scaffold RNA (ScRNA) able to recruit a variable number of VP64 activation domains. The activator arises from the complex between the synthetic ScRNA and DNase-deficient type V Cas proteins: dCas12e and denAsCas12a. The transcription activation achieved via the newly engineered dCas:ScRNA system is up to 4.7-fold higher than that obtained with the direct fusion of VP64 to Cas proteins. The new transcription factors have been proven to be functional in circuits such as Boolean gates, converters, multiplex-gene and metabolic-pathway activation. Our results extend the CRISPR-Cas-based technology with a new effective tool that only demands RNA engineering and improves the current design of transcription factors based on type V Cas proteins.

## Graphical Abstract

**Graphical Abstract:**
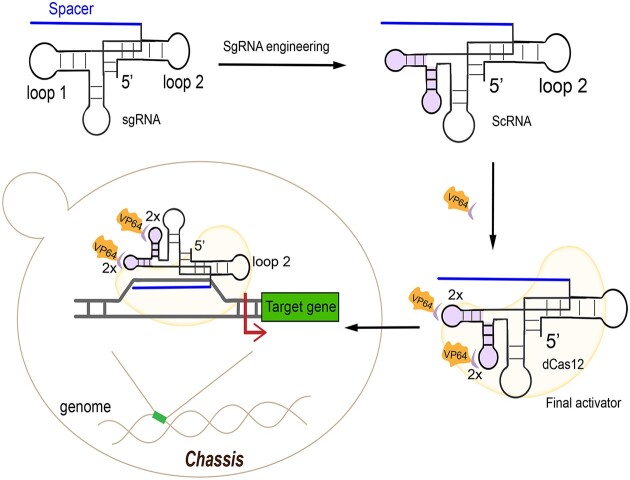

## Introduction

The CRISPR-Cas system, an RNA-based component of the immune system of bacteria and archaea, has become rapidly a widely used tool for genome editing and gene regulation in both prokaryotic and eukaryotic cells. In class 2 CRISPR-Cas systems (including type II, type V and type VI), DNA or RNA cleavage is carried out by a single CRISPR associated protein (Cas9, Cas12 and Cas13, respectively) upon forming a complex with a mature guide RNA sequence—termed either CRISPR RNA (crRNA) or single guide RNA (sgRNA)—complementary to the target DNA or RNA (1). Gene regulation is achieved by engineering nuclease-deficient Cas proteins (dCas). (d)Cas12a, which is representative of type V CRISPR-Cas system (2), makes complex with a short crRNA (∼40 nt) that consists of a direct repeat (DR) and a spacer (sp). Differently from type II CRISPR-Cas9, the transactivating RNA (tracrRNA) is not required (3–5). Cas12a has a double-nuclease activity because it cuts both RNA and DNA sequences. Hence, (d)Cas12a processes its precursor CRISPR-RNA (pre-crRNA) without the help of any endoribonucleases (e.g. RNase III), which simplifies its application to multiplex gene editing or regulation (6,7). Every DNase-deficient Cas protein is turned into a transcriptional factor (TF) upon fusion to an activation (e.g. VP64 and VPR) or a repression domain (e.g. Mxi1 and KRAB). dAsCas12a (isolated from the bacterium *Acidaminococcus* sp.*BV3L6*) was used as both a repressor and an activator in human, plant, and yeast cells (8–10). Moreover, an activator built on dLbCas12a (from *Lachnospiraceae bacterium*) was a component of a biosensor for the detection of tumor-related signals in mammalian cells (11). In 2021, Xu *et al.* realized and tested in mammalian cells a new compact activator, called dCasMINI-VPR, based on a mutant of Cas12f (12).

Cas12e—previously called CasX—is another type V CRISPR-associated protein that was isolated from *Planctomycetes* and first reported in 2017 (13). Cas12e is compact and small-sized: <1000 AA, whereas other popular Cas proteins such as AsCas12a, LbCas12a and SpCas9 are made of about 1200 AA. Among type V Cas proteins, Cas12e displays some unique features (14,15). First, Cas12e recognizes 5′-TTCN as a protospacer adjacent motif (PAM) instead of 5′-TTTV (V means not T), the sequence detected by Cas12a. Second, Cas12e makes a complex with a single-guide RNA (sgRNA)—like Cas9—rather than a mature crRNA, as in Cas12a case. Cas12e sgRNA is longer than Cas12a crRNA and the presence of a tracrRNA provides more engineering opportunities (16). In 2019, Liu *et al.* found that the bare DNase-deficient Cas12e (dCas12e) could silence GFP (green fluorescent protein) expression in *E. coli* (14). In 2021, the chimeric activator dCas12e-VPR and repressor dCas12e-KRAB were characterized in human cells by Cao *et al.* (17).

These studies showed how to engineer new, working TFs by taking dCas12a and dCas12e as templates to which activation/repression domains (dCas12-AD/RD) were fused. However, the CRISPR-dCas9 system had previously led to the construction of highly performant TFs by turning the sgRNA into a scaffold RNA (ScRNA) where domains of diverse typology were added or recruited. The ScRNA approach appeared to guarantee higher control of gene expression than the chimeric dCas9 in mammalian cells, *Saccharomyces cerevisiae*, and plants (7,16,18–21).

In this work, we have realized a new activation system, termed dCas12:ScRNA, by engineering scaffold RNAs for dCas12e and denAsCas12a proteins. To this aim, we modified the original type V sgRNA/crRNAs with the insertion of a variable number of RNA hairpins able to bind small coat proteins where the AD was fused. dCas12:ScRNA was tested successfully as a component of synthetic gene circuits such as Boolean gates and converters, and in multiplex-gene and β-carotene metabolic-pathway activation. Overall, our results show that dCas12:ScRNA enhances protein synthesis far more than the dCas12-AD:sgRNA/crRNA complex, which was obtained by fusing the AD to dCas12 directly, without any modification in the sgRNA/crRNA. These results resemble those that we published about dCas9-based activators (20).

## Materials and methods

### Plasmid construction

The plasmids constructed in this work were based on the integrative yeast shuttle vectors pRSII40X (X represents an auxotrophic marker)—a gift from Steven Haase (22)—obtained from Addgene. The list of all plasmids assembled in this work is given in Supplementary Material Excel Sheet 1.

The DNA sequences of the proteins used in this work were yeast-codon optimized and synthesized by Genewiz Inc., Suzhou (China). They are: dCas12e, denAsCas12a, dLbCas12a, MCP-VP64, PCP-VP64, QCP-VP64, Com-VP64, yEGFP, yEBFP, ABI, PYL1, GID1, GAI, Flex-loxp, MCP-P65-HSP1, crtE-Tsynth24, crtI-Tsynth6 and crtYB-TGUO1. The protein recombinase (Cre) was a gift from Yingjin Yuan (Tianjin University).

#### Construction of plasmids containing a fluorescent protein

The DNA sequences of the two fluorescent proteins—yEGFP and yEBFP—were first amplified via PCR from template plasmids. These coding regions (CDSs) were then incorporated, together with a promoter (either the synthetic promoter 1x/3x_lexOpR/tetOp_truncated_pCYC1core or the constitutive *GPD/REV1* promoter) and the synthetic terminator Tsynth24 (23), into the cut-open vector pRSII405 (digested with Acc65I (NEB-R0599S) and SacI-HF (NEB-R3156S)) via Gibson assembly (24).

#### Construction of plasmids containing a Cas protein

BamHI-dCas12e/denAsCas12a/dLbCas12a-Xhol fragments were amplified via PCR from the corresponding template plasmids and inserted into acceptor vectors designed *ad hoc*—pRSII406-pGPD-ATG-NLS-GS-HIStag-GS-BamHI-sp-XhoI-(nothing/-linker_VPR/_VP64/_Mxi1)-NLS-TAA-TGUO1, where ‘sp’ represents a short random sequences, and ‘nothing’ means that the bare protein was used—via Gibson assembly. Both vector and Cas-template plasmid were digested with BamHI (NEB-R0136V) and XhoI (NEB-R0146V). The insert (Cas protein) was then ligated to the cut-open vector with T4 DNA ligase (NEB-M0202S—over 16 h at 16°C).

#### Construction of plasmids containing a coat protein

The sequences Xhol-NLS-MCP/PCP/QCP/Com-VP64 or Xhol-NLS-MCP-P65-HSP1 were obtained from template plasmids via PCR and then assembled, together with pADH1 and ADH1t, into a cut-open vector (pRSII404, digested with Acc65I and SacI-HF) via Gibson method give rise to the P1 plasmids: pRSII404-pADH1-Xhol-NLS-(MCP/PCP/QCP/Com-VP64)/(MCP-P65-HSP1)-ADH1t-SacI. P1 plasmids were used as acceptor vectors for the construction of two more kinds of plasmids: pRSII404-pADH1-Xhol-NLS-MCP-VP64-linker_VP64-TGUO1-SacI and pRSII404-pADH1-Xhol-NLS-MCP/PCP/QCP-Mxi1-CYC1t-SacI. In these cases, MCP-VP64 (MCP) and linker_VP64-TGUO1 (Mxi1-CYC1t) were obtained via PCR and then assembled, via Gibson method, together with a cut-open P1 plasmid (digested with Xhol and SacI-HF).

#### Construction of plasmids containing hetero-dimerization proteins

Two acceptor vectors: pRSII403-pADH1-NLS-MCP-BamHI-sp-XhoI-FLAG_tag-TAA-ADH1t and pRSII405-pTEF2-HA_tag-BamHI-sp-XhoI-linker_VP64-NLS-TGUO1 together with the expression plasmid pRSII406-pGPD-ATG-NLS-GS-HIStag-GS-BamHI-dCas12e-XhoI-GS-NLS-TAA-CYC1t-SacI were, initially, assembled via Gibson method. Then, the fragments BamHI-ABI/PYL1/GID1/GAI-XhoI or Xhol-ABI/PYL1/GAI-Xhol-TAA-ADH1t-SacI were incorporated inside the cut-open plasmids (digested with BamHI and XhoI or Xhol and SacI) via Gibson method again.

#### Construction of plasmids containing the ScRNA expression cassettes

dCas12e sgRNA sequences were based on (14), whereas the crRNA for denAsCas12a and dLbCas12a came from our previous work (10). ScRNAs were built via PCR. The full plasmids expressing ScRNAs were assembled via Gibson method. Information about ScRNA design and construction are given in the ‘ScRNA’ section of the Supplementary Material.

#### Construction of plasmids containing the β-carotene pathway components

The acceptor vector pRSII405-Acc65I-pTEF2-HAtag-BamHI-sp-XhoI-linker_VP64-NLS-TGUO1-SacI was constructed via Gibson method. crtE-Tsynth24, obtained from a template plasmid via PCR, was inserted, together with pTEF1-SalI, into the above cut-open vector (digested with Acc65I and BamHI) via Gibson assembly to get plasmid 1: pRSII405-Acc65I-pTEF1-SalI-crtE-Tsynth24-BamHI-sp-XhoI-linker_VP64-NLS-TGUO1-SacI. Afterwards, plasmid 1 was cut-open with Xhol and SacI to incorporate, via Gibson method, crtI-Tsynth6, together with 3xlexOpR_trunc_pCYC1core, and obtain plasmid 2: pRSII405-Acc65I-pTEF1-SalI-crtE-Tsynth24-BamHI-sp-XhoI-3xlexOpR_trunc_pCYC1core-crtI-Tsynth6-SacI. Finally, plasmid 2 was cut-opened with BamHI and Xhol to replace the random spacer ‘sp’ with the 3xtetOp_trunc_pCYC1core and crtYB-TGUO1 fragment (obtained by PCR) and construct the three-gene-expression plasmid 3: pRSII405-Acc65I-pTEF1-SalI-crtE-Tsynth24-BamHI-3xtetOp_trunc_pCYC1-crtYB-TGUO1-XhoI-3xlexOpR_trunc_pCYC1core-crtI-Tsynth6-SacI. A plasmid where crtE was controlled by pREV1 was obtained by using plasmid 3 as cut-open vector (digested with Acc651 and SalI (NEB-R0138V)) where the *REV1* promoter was inserted via Gibson method.

All PCRs were touchdown PCRs. They made use of Q5 High-Fidelity DNA Polymerase (NEB-M0491S). DNA fragments extracted from PCR products were purified via the AxyPrep DNA extraction kit (Axigen-AP-GX-250). 5 μl DNA parts (both PCR products and the cut-open vector) were added into 15 μl Gibson Isothermal assembly mixture and reacted at 50°C for 1 h. 50 μl DH5α (Life Technology––18263-012) *Escherichia coli* cells were transformed, via a short heat shock at 42°C, with 5 μl of the solution containing the new assembled plasmids (10).

### Yeast transformation

*S. cerevisiae* strain CEN.PK2-1C (MATa; his3Δ1; leu2-3_112; ura3-52; trp1-289; MAL2-8c; SUC2), Euroscarf-30000A (Johann Wolf-gang Goethe University, Frankfurt, Germany)—termed byMM584 was used as a chassis for all circuits constructed in this work. Lithium acetate-thermal shock yeast transformation (25,26) with integrative plasmids demanded to linearize 5 μg of plasmids via digestion inside the auxotrophic marker. Lithium acetate, polyethylene glycol (PEG), dimethyl sulfoxide (DMSO), and salmon sperm DNA were used to facilitate the uptake of plasmids by the cells. The heat shock lasted 15 min at 42°C. After transformation, yeast cells grew on solid synthetic selective media (2% glucose, 2% agar) for up to 4 days at 30°C. All strains used/realized in this work are listed in Supplementary Material Excel sheet 2.

### Cell culture and media

Media for yeast culture, i.e. YPD rich medium, synthetic defined complete medium (SDC), SD selective media, and SDC supplied with 10 mM methionine (CAS number: 63-68-3) were prepared according to the details in (26). 50 mM stock solutions of GA3 (CAS number: 77-06-5) and ABA (CAS number: 21293-29-8) were prepared for GA3 and ABA titration curves (YES and AND gate testing). 50 mM GA3 (ABA): dissolve 20.9 (13.22) mg GA3 (ABA) in 1 ml Ethanol (Analytical grade) and sterilize it with a 0.22 μm filter membrane (avoid using artificial light during the whole process).

### Flow cytometry

Strains were cultured, first, at 30°C 240 RPM in 2 ml SDC or SDC supplied with methionine (10 mM), GA3 or ABA for 16 h. Then, cell solutions were diluted (1:20) into the same medium to grow for 8 more hours. 20 μl cell culture were then added to 300 μl ddH2O. BD FACSVerse machine was used for fluorescence intensity evaluation (laser 488 nm-FITC filter 527/32 nm, and laser 405 nm-Pacific Blue filter 448/45 nm were selected for green and blue fluorescence detection, respectively). To gain reliable data, we checked the performance quality control (PQC) of the machine and used fluorescent beads (BD FACSuite CS&T Research beads 650621) to regulate the voltage with FITC and Pacific Blue filter. The criterion to adjust the voltage is that the difference of the peak values between two consecutive bead measurements should not be bigger than 5%. Ten thousand cells were collected in each independent experiment (three at least) and fluorescence mean value—analyzed with the flowcore R-Bioconductor package (27)—was utilized for data analysis and comparison. Statistical analyses were based on two-sided Welch's *t* test and one-way ANOVA (for all FACS analyses, see Supplementary Tables S1–S23 and S26–S27 in the section ‘Supplementary Tables with Statistical Data Analysis’ and Supplementary Material Excel sheet 3).

### RT-qPCR

RNA extraction from *S. cerevisiae* and cDNA synthesis were carried out via YeaStar RNA kit (Zyomo Research-R1002) and HiFiScript cDNA Synthesis kit (CWBIO-CW2569M). As for qPCR, we utilized 2xSYBR Green qPCR Mix kit (AH0104, Shandong Sparkjade Biotechnology Co., Ltd). *ACT1* was taken as a reference gene. Primers for *yEGFP*, *yEBFP* and *ACT1* genes were designed with the Primer3Plus (28) program, those for *HED1*, *GAL7*, and *CYC1* were taken from references (29,30). 10 μl reaction solution was prepared according to the instructions in the 2xSYBR Green qPCR Mix kit and analyzed with a Roche LightCycler96 machine with a two-step program: (i) hold stage, 10 min at 95°C; (ii) amplification step: 15 s at 95°C followed by 34 s at 55°C (40 cycles); (iii) melting curve stage: 10 s at 95°C, then 60 s at 65°C, and, finally, 1 s at 97°C. Each sample was measured at least three times. Gene amplification was calculated via the Pfaffl formula (31) (all primers used in qPCR and more details on the endogenous target sequence are available in Supplementary Material, section ‘RT-qPCR’; numerical results are shown in Supplementary Tables S24–S25 and S28–S31).

### Characterization of β-carotene

Transformation plates for the whole β-carotene metabolic pathway with and without the dCas12:ScRNA activators were incubated at 30°C for 6 days. Then, single colonies were transferred to fresh plates where they grew for two more days. Cells were taken with a sterile loop and diluted in SDC solution to get OD equal to 1.0. 10 μl of each cell solution were dropped into a new plate. This plate was put into a 30°C incubator for 6 days. Finally, the plate was photographed with a NIKON DIGITAL CAMERA D3200.

### Yeast samples preparation for RNA-seq

Yeast strains grew overnight before being diluted (1:20) into fresh solution. Then, they grew for 8 more hours at 30°C, 240 RPM. Cells were then centrifuged at 3000 × g for 5 min. The supernatant was discarded and the cells were frozen with liquid nitrogen.

## Results

### Construction of dCas12e-based activators in *S. cerevisiae*

To turn dCas12e:sgRNA into an *S. cerevisiae* activator, we initially fused an activation domain (either VP64 or VPR) to dCas12e (see Supplementary Figure S1A-B). To detect activation easily, we adopted a synthetic weak promoter (termed ‘truncated_pCYC1core’—see Supplementary Table S1 for a comparison with native promoters in *S. cerevisiae*) to control the expression of the yeast enhanced green fluorescent protein (yEGFP) (10). The truncated_pCYC1core harbors one (1×) or three (3×) lexOpRs—i.e. the right part of the full lex operator (32)—upstream of a variant of the *CYC1* core promoter where the 5′UTR (untranslated region) was reduced from 71 to 24 bp. Two sgRNAs were tested in this circuit, one binding the anti-sense strand (bA) of lexOpR, the other the sense strand (bS). dCas12e-VP64/VPR:sgRNA showed activation efficiency close to that of dLbCas12a- and denAsCas12a-based activators (10) (see Supplementary Figure S1C). The highest fluorescence enhancement (3.52-fold) with respect to the control circuit (in which the sgRNA was not expressed), was reached by dCas12e-VPR:sgRNA acting on the sense strand of 3xlexOpR. Different from dCas12a-based activators (10), dCas12e-VP64/VPR:sgRNA induced higher gene expression when targeting lexOpR on the sense strand. In most cases, the sgRNA binding the anti-sense strand failed to increase fluorescence significantly with respect to the control circuit (see in Supplementary Table S2). Hence, in the next experiments, only sgRNAs targeting the sense strand were used.

We focused, then, on the construction of a new kind of activator where the ADs were recruited by a modified sgRNA, i.e. the scaffold RNA. In order to link sgRNA and ADs, we resorted to, as in (18), RNA hairpin—coat protein pairs made of a long, viral, non-coding RNA that folds into a hairpin secondary structure recognized and bound by a unique coat protein. The RNA hairpin—coat protein pair becomes a bridge between the sgRNA and the AD after fusing the sgRNA to the RNA hairpin and the AD to the corresponding coat protein. It was crucial, therefore, to identify the regions of the sgRNA that could be modified with a viral RNA hairpin without compromising the formation of the dCas12e:sgRNA complex. We analyzed the three-dimensional structure of Cas12e:sgRNA (14) and identified three favorable positions: the 5′-end, loop 1 and loop2 (see Supplementary Figure S1D). Loop 1 is far from the core of the Cas12e:sgRNA complex; loop 2 and the 5′-end look like ‘exposed’ peripherical motifs rather than embedded in the structure of the complex.

**Figure 1.**
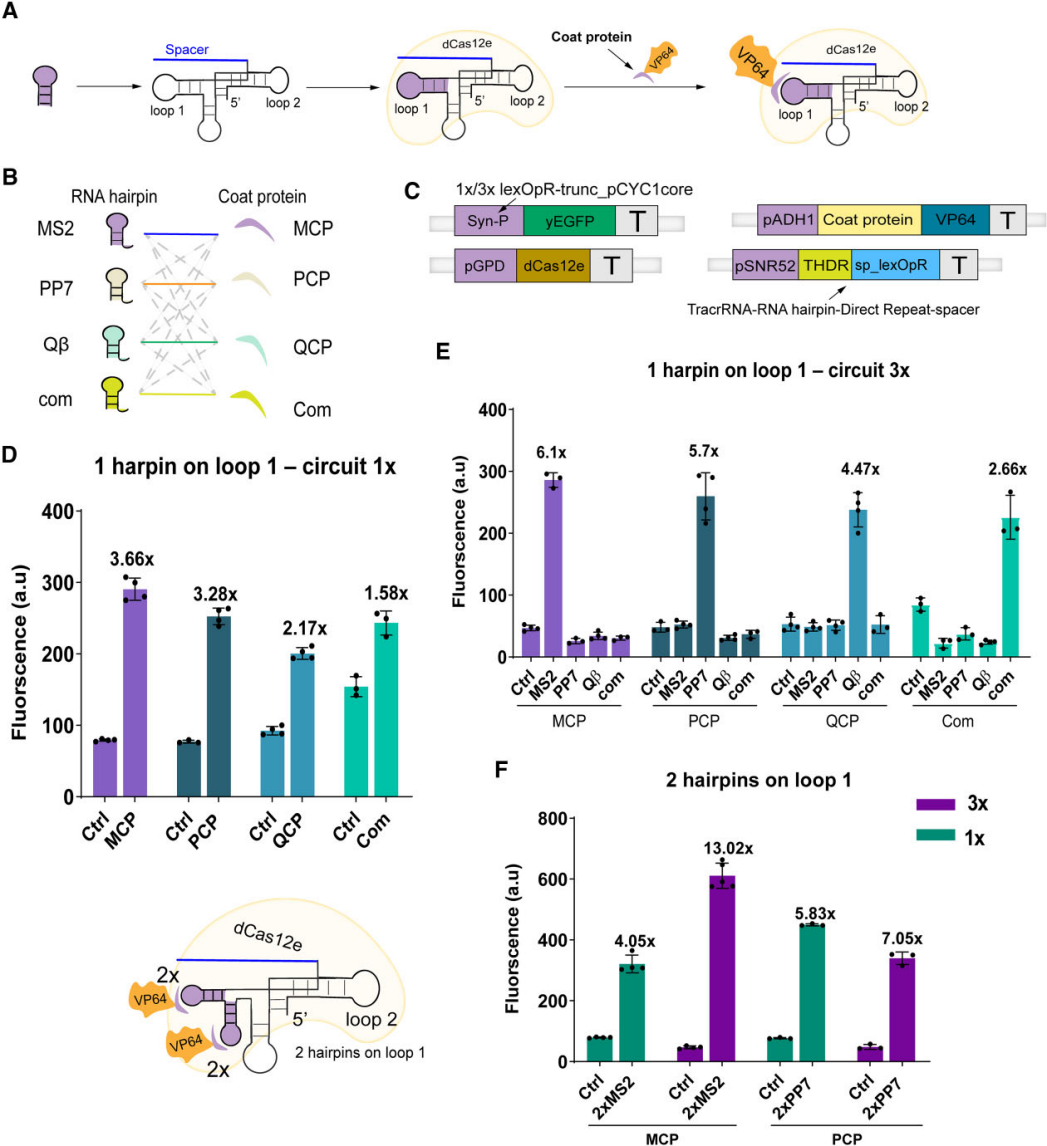
New *S. cerevisiae* activators based on the interaction between dCas12e and its scaffold RNA. (**A**) The (d)Cas12e single-guide RNA is turned into a scaffold RNA by inserting one hairpin on loop 1. The complex dCas12e:ScRNA behaves like an activator since the RNA hairpin recruits specific coat proteins fused to the VP64 AD. (**B**) The four RNA hairpin—coat protein pairs (MS2-MCP, PP7-PCP, Qβ-QCP, and com-Com) used in this work and all possible combinations among them. (**C**) Activation circuits. The reporter protein, yEGFP, is controlled by a synthetic weak promoter (Syn-P) (10) that hosts either a single or three lexOpRs. dCas12e and the chimeric protein resulting by the fusion of a coat protein with VP64 are constitutively expressed by strong promoters. The ScRNA contains the original tracrRNA (T) followed by loop 1 (modified with the insertion of one or two hairpins—H), the direct repeat (DR), and the spacer complementary to lexOpR (**sp**_lexOpR). ScRNA synthesis demands an RNA polymerase III-dependent promoter (pSNR52). **(D)** The activation efficiency of dCas12e:ScRNA on circuit 1x (i.e. Syn-P hosts a single lexOpR) by varying the four RNA hairpin—coat protein systems. Loop 1 contains a single hairpin. (**E**) Activation efficiency of dCas12e:ScRNA on circuit 3× with the four RNA hairpin—coat protein pairs. The orthogonality among the four RNA hairpin—coat protein pairs is apparent since fluorescence is enhanced only when a coat protein interacts with its corresponding RNA hairpin. (**F**) Schematic and activation efficiency of dCas12e:ScRNA when loop 1 hosts a double hairpin (only MS2-MCP and PP7-PCP pair were tested). Each hairpin recruits two copies (2×) of the corresponding coat protein linked to VP64. Values on the top of the bars refer to the activation folds with respect to the control circuits (‘Ctrl’) in which the ScRNA was absent.

Initially, we selected loop 1 to insert the RNA hairpins (see Figure 1A) and assess the working of four RNA hairpin—coat protein pairs (MS2-MCP, PP7-PCP, Qβ-QCP, and com-Com, see Figure 1B) (33). Previously, Pan *et al.* succeeded in constructing a synthetic activator in plant (termed dAaCas12b-Act3.0) by incorporating the MS2 aptamer into the sgRNA of dAaCas12b (7), which hinted to the possibility of extending type V mature crRNA as a ScRNA.

We placed a single RNA hairpin on loop 1 and fused VP64 to the coat protein. The ScRNA induced a significant activation on the two circuits that we constructed (they are indicated as 1× and 3× since they differ only for the number of lexOpR in the synthetic promoter upstream of *yEGFP*—see Figure 1C). MS2-MCP was the most performant pair (3.66-fold in 1× circuit (*P*-value < 0.0001, *n* = 4—number of replicates), 6.1-fold in 3× circuit (*P*-value = 0.0003, *n* = 3)), whereas com-Com was the only pair that failed to reach the ‘working threshold’ (2-fold) (34) when acting on a single lexOpR. It should be noted, though, that the low activation efficiency of Com-com was due only to its high basal expression (i.e. the fluorescence level of the control circuit) and not to a low activation induced by the complete system. Three lexOpRs guaranteed, as expected, a higher ON/OFF ratio than a single lexOpR (see Figure 1D, E and Supplementary Tables S3–S4).

In addition, we studied the orthogonality among the four RNA hairpin—coat protein pairs by testing all 16 possible combinations of the coat proteins and the hairpin structures. The results, shown in Figure 1E, point out that each RNA hairpin recruits its own coat protein only and does not interact with the other three, which means that there is no crosstalk between the four RNA hairpin—coat protein pairs. Therefore, more RNA hairpin—coat protein pairs can be used within the same circuit (see in Supplementary Table S4).

Next, we added to loop 1 a double RNA hairpin (either 2× MS2 or 2× PP7, each recruiting two coat proteins fused to VP64) (18). The new ScRNA improved the ON/OFF ratio on both 1× and 3× circuit, with the highest value (13.02, *P*-value < 0.0001, *n* = 5) achieved by the MCP-MS2 pair in the 3× circuit (see Figure 1F and Supplementary Figure S1E, Supplementary Table S5).

On the whole, our results point out that loop 1 is an advantageous position to engineer a scaffold RNA for (d)Cas12e. The insertion of RNA hairpins that interact with specific coat proteins fused to a strong AD led to the construction of new activators. Therefore, loop 1 structure can be modified without preventing the formation of the dCas12e:ScRNA complex and its binding to the DNA. The MS2-MCP RNA hairpin—coat protein pair appeared to be the most performant among the four we tested. The usage of two hairpins on loop 1 allowed recruiting more MCP-VP64 with a consequent increase in fluorescence expression.

### Evaluating more configurations of the dCas12e:ScRNA complex

**Figure 2.**
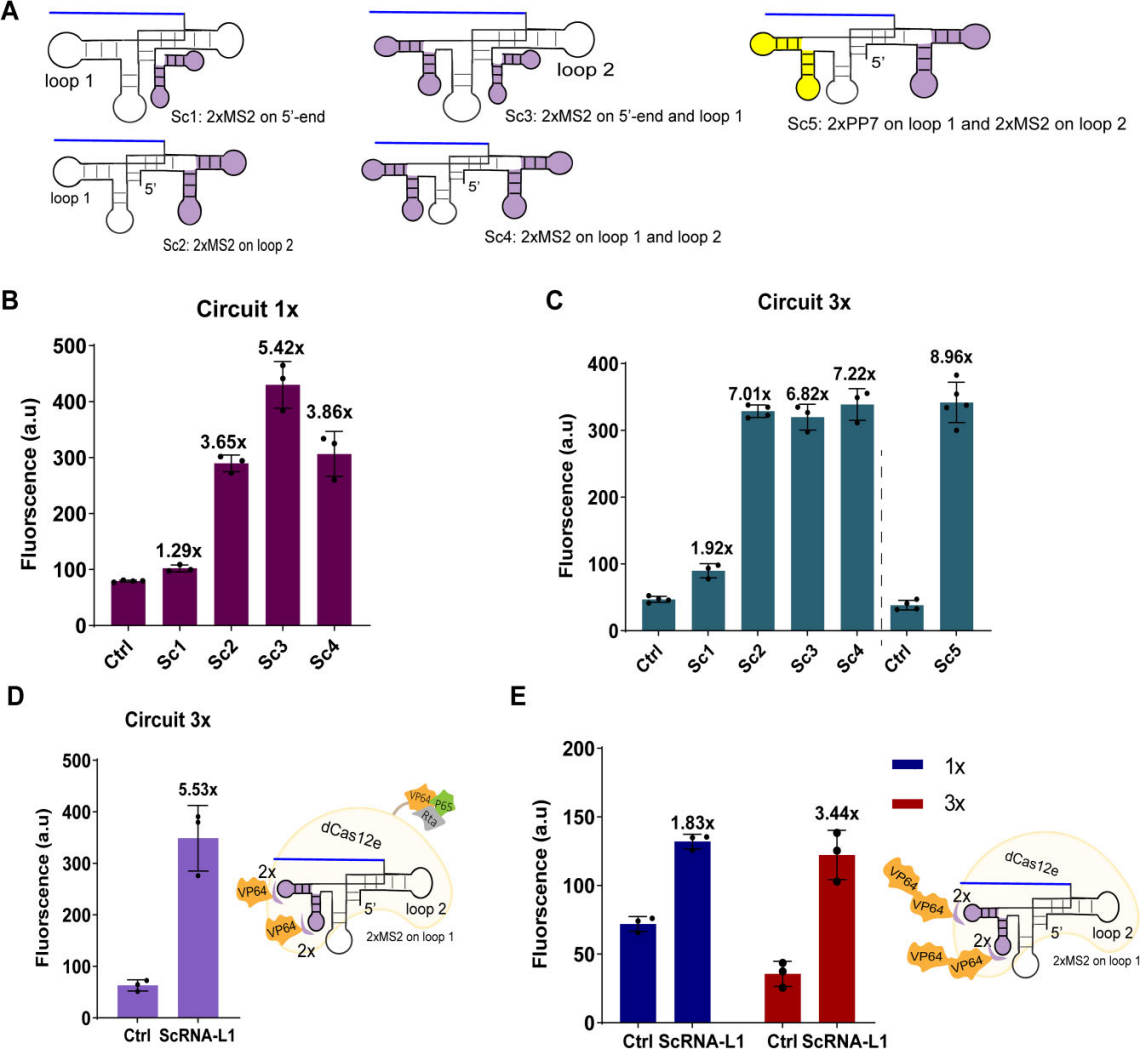
Comparing different configurations of the dCas12e:ScRNA complex. (**A**) ScRNA structures. They were constructed by inserting one or two double RNA hairpins (2× MS2—in purple—or 2× PP7—in yellow) into one or two of the three selected regions (loop 1, loop 2, and 5′-end) of dCas12e sgRNA. (B, C) Activation efficiency of dCas12e:ScRNA variants in the circuits with one (**B**) and three lex operators (**C**). (**D**) The activation efficiency of dCas12e-VPR:ScRNA in the 3× circuit. (**E**) Fluorescence ON/OFF ratios in the circuits hosting the dCas12e:ScRNA variant with 2× VP64 fused to MCP. Values on the top of bars refer to the activation folds with respect to control circuits, ‘Ctrl’, where the ScRNA is not expressed.

Besides loop 1, we inserted a double MS2 hairpin onto two more regions (the 5′-end and loop 2). Moreover, we constructed three more ScRNA configurations where a double RNA hairpin was inserted into two different positions, namely: 5′-end and loop 1 (2xMS2 on each location); loop 1 and loop 2 (2× MS2 on each loop); and loop1 and loop2 (2× PP7 on loop 1 and 2× MS2 on loop 2—see Figure 2A and Supplementary Figure S2A). Our results show that, in circuit 1×, only by placing 2× MS2 on both 5′-end and loop 1 the ON/OFF ratio increased with respect to the case of 2× MS2 on the sole loop 1 (5.42 versus 4.05, see Figure 2B and Supplementary Table S6). In contrast, the activation efficiency of the other new ScRNA configurations did not exceed that due to a double MS2 hairpin on loop 1, no matter how many operators were present on Syn-P. The highest ON/OFF ratio was, nevertheless, a significant 8.96 (*P*-value < 0.0001, *n* = 5, see Figure 2C and Supplementary Table S7). The configuration with a double RNA hairpin on the 5′-end only led to a low activation (<2-fold) in both 1× and 3× circuit (see Figure 2B and C).

On the whole, loop 1 appears to be the best location for engineering Cas12e sgRNA. In particular, the most performant ScRNAs demanded MS2 (in a single or double copy) on loop 1.

For a comparison with a well-studied activation system, we built three circuits containing dSpCas9:ScRNA acting on either 1× lexOpR or 3× lexOpR upstream of the truncated_pCYC1core. dSpCas9-based activators—where ScRNA hosted 2× MS2 on a single location—turned out to be fairly stronger than dCas12e:ScRNA on both 1× (2.08-fold) and 3× (1.56-fold) circuit. In the presence of a double hairpin on two different locations of the scaffold RNA, dSpCas9:ScRNA became up to 3.5-fold stronger than dCas12e:ScRNA (see Supplementary Figure S2B). From this comparison, we can conclude that dCas12e, although less performant, is nevertheless a reasonable alternative to dSpCas9 for the construction of activators based on scaffold RNAs.

We made two more attempts to further enhance the performance of dCas12e-based activators. The first one combined dCas12e direct modifications with sgRNA engineering (see Figure 2D and Supplementary Figure S2C). Here, we expressed the chimeric protein dCas12e-VPR together with an ScRNA hosting 2× MS2 on loop1. In the second test, we fused two copies of VP64 to the MCP coat protein (see Figure 2E and Supplementary Figure S2D). Both new configurations did not provide a better activation than that of the previous designs (see Figure 2D, E and Supplementary Tables S8–S9).

Beside activation, we also studied repression of transcription, in *S. cerevisia*e, by dCas12e-based systems. We engineered two kinds of repressors—dCas12e(-Mxi1):sgRNA and dCas12e:ScRNA-Mxi1—that targeted the yeast enhanced blue fluorescent protein (yEBFP) (35). Moreover, we constructed dLbCas12a-based repressors as a positive control (10). Both variants of dCas12e-based repressors failed to decrease gene expression (see Supplementary Figure S2F) in striking contrast with dLbCas12a-Mxi1:crRNA that reduced fluorescence up to 81% (see Supplementary Figure S2E).

Taken together, we have shown that a new type of activator is realized by turning Cas12e sgRNA into a scaffold RNA that recruits multiple copies of VP64. Similar to dCas9:ScRNA, also dCas12e:ScRNA shows higher activation efficiency than that reached by fusing the AD to the Cas protein directly. The strongest configuration that we engineered came from the combination of the bare dCas12e with an ScRNA hosting 2× MS2 on loop 1. Thus, in the following experiments we employed only this highly performant activation system that is referred to as dCas12e:ScRNA-L1.

### Engineering a highly effective denAsCas12a ScRNA by placing 2× MS2 at the modified 5′-end of the crRNA

**Figure 3.**
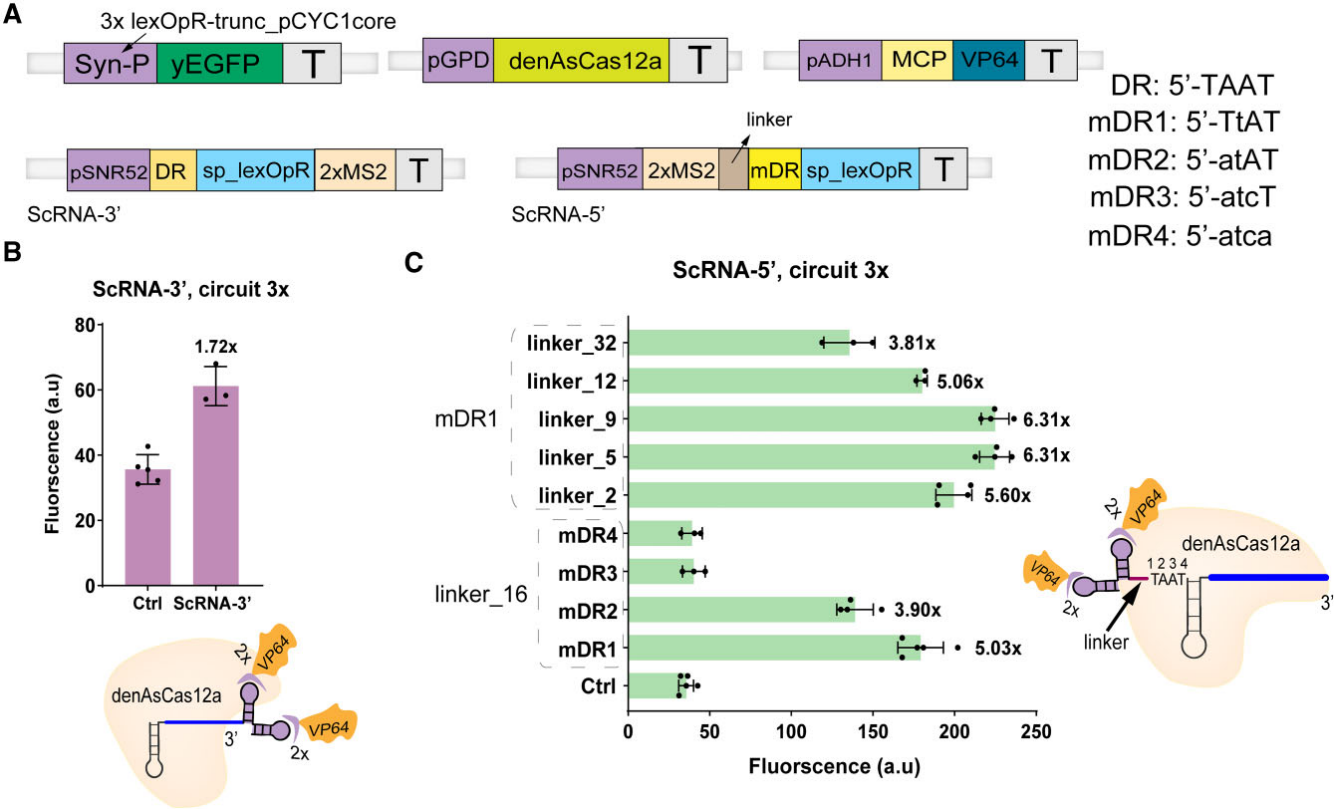
Engineering and optimizing the ScRNA for denAsCas12a. (**A**) The transcription units in the circuits hosting denAsCas12a:ScRNA activators. The 2× MS2 motif was inserted into either the 3′- or 5′-end of denAsCas12a crRNA to build ScRNA-3′ and ScRNA-5′, respectively. Four initial versions of ScRNA-5′ were constructed by mutating the DR (mDR1 to mDR4). (**B**, **C**) Activation efficiency of denAsCas12a:ScRNA-3′ and denAsCas12a:ScRNA-5′, respectively. In (C), the four mutated direct repeats (mDR1 up to mDR4) were first tested in association with a 16-bp-long linker. The length of the linker was, then, changed from 2 to 32 bp by keeping mDR1 fixed in the ScRNA-5′ structure. ‘Ctrl’ is a circuit without ScRNA expression. Values on top of bars are the activation enhancements with respect to ‘Ctrl’.

In a previous study, we showed that denAsCas12a-AD:crRNA was a good activator in *S. cerevisiae* (10). Considering the above results obtained for CRISPR-dCas12e, we investigated if an ScRNA could be implemented in the CRISPR-denAsCas12a system too. Initially, we placed the 2× MS2 hairpin on the 3′-end of denAsCas12a crRNA (ScRNA-3′, see Figure 3A for the activation circuit). However, as shown in Figure 3B and Supplementary Table S10, denAsCas12a in complex with ScRNA-3′ induced a low activation on the 3× circuit (only 1.72-fold, *P*-value = 0.0052, *n* = 3). We thought that 2× MS2 in 3′-end might interfere with the binding of the spacer to the target DNA. Therefore, we moved 2× MS2 to the 5′-end of the crRNA (ScRNA-5′). Here, we had to mutate the DR sequence to prevent the loss of 2× MS2 due to the RNase activity of denAsCas12a. We tried four different mutant versions of DR (named mDR1 to mDR4) and introduced a 16-bp-long linker between 2× MS2 and the mDRs (see Figure 3C and Supplementary Table S11). mDR1 and mDR2 displayed higher activation than 2-fold. In particular, mDR1 provoked a 5.03 ON/OFF ratio (*P*-value < 0.0001, *n* = 5).

We further modified ScRNA-5′ by keeping mDR1 and changing the length of the linker from 2 to 32 bp. Short linkers (5 and 9 bp) assured an activation increase up to 6.31-fold (*P*-value < 0.0001, *n* = 4), which is almost 2.69 times higher than that obtained by fusing VP64 to denAsCas12a directly (2.35-fold, *P*-value 0.0002, *n* = 3, see Supplementary Figure S3A and Supplementary Table S12). Longer linkers appeared to decrease activation efficiency. This became particularly evident with the 32-bp-long linker because the ON/OFF ratio dropped to 3.81 (*P*-value = 0.0060, *n* = 3).

We tested also the P65-HSF1 AD, already employed in mammalian cells (16), together with dCas12e:ScRNA-L1 and denAsCas12a:ScRNA-5′ structure. However, it turned out to be unfunctional in *S. cerevisiae*—see Supplementary Figure S3B and Supplementary Table S13.

These experiments show that an ScRNA can be built for denAsCas12a as well. Moreover, it permits to accomplish higher activation than by modifying denAsCas12a directly, as it happened with dCas12e and dCas9 too. The optimal ScRNA configurations for the CRISPR-denAsCas12a system contain the mutated direct-repeat mDR1 and a short (5 or 9 bp) linker. In the rest of this work, we adopted only the configuration with the 5-bp-long linker that will be indicated just as ScRNA-5′.

### Plant hormone inducible systems based on the dCas12e:ScRNA activator

**Figure 4.**
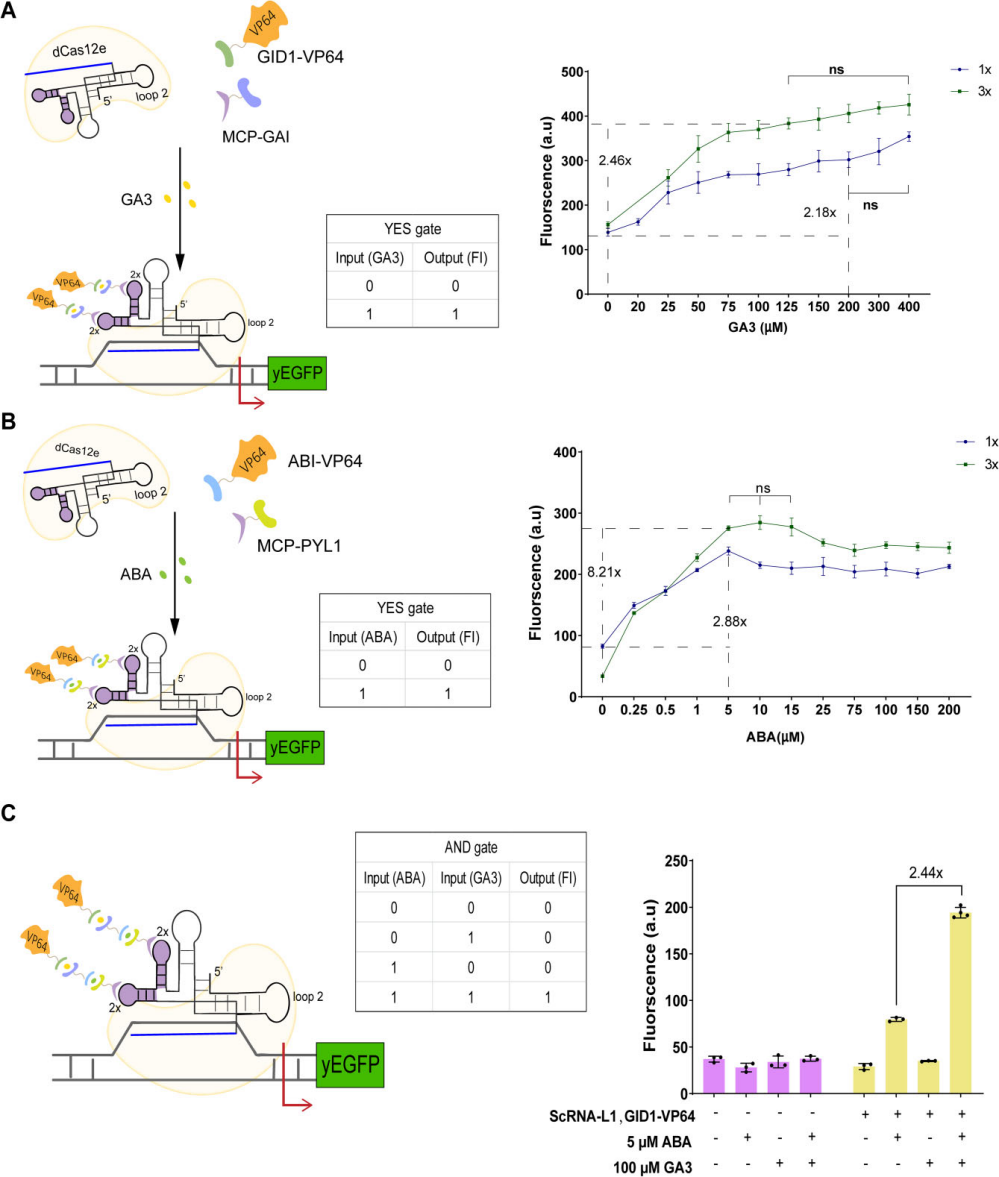
Plant hormone inducible systems based on the dCas12e:ScRNA-L1 activator. (**A**) YES gate responding to gibberellin (GA3). Left panel: GA3 induces the GID1–GAI hetero-dimerization process that leads to the formation of the activator responsible for increasing yEGFP expression. Right panel: fluorescence as a function of GA3 concentration in both 1× and 3× circuit. (**B**) YES gate hosting the hetero-dimerization system (PYL1-ABI) controlled by abscisic acid (ABA). PYL1 and ABI were fused to MCP and VP64, respectively. The two domains do not interact until ABA is present in the circuit. Both 1× and 3× circuit reached the highest activation at 5 μM ABA. (**C**) AND gate. The output is expected to be ‘1′ only in the presence of both ABA and GA3. However, a slight increase in yEGFP expression is detected also in the presence of the only ABA. ‘ns’ (no statistical difference), in (A) and (B), corresponded to *P*-value >0.05 (one-way ANOVA). The value on the top of the line in (C) refers to the AND gate ON/OFF ratio calculated between the only ‘1′ and the maximal ‘0′ outputs.

Since the inputs of a large number of synthetic gene circuits are chemicals (36), we tested the working of dCas12e:ScRNA-L1 as an inducible activator by coupling it with plant hormone-inducible hetero-dimerization systems. Initially, we engineered Boolean gates responding to a single input (YES or buffer gates). As a first input, we used the plant hormone gibberellin (GA3) that triggers the hetero-dimerization between GID1 and GAI domains (37,38). GAI was fused to MCP, GID1 to the VP64 (see Supplementary Figure S4A-Design 1). In the presence of GA3, the dimerization of GID1 with GAI established a link between MCP and VP64. Once MCP had bound ScRNA-L1, a complete dCas12e:ScRNA-L1 activator was formed (see Figure 4A and Supplementary Table S14). The expression level of yEGFP increased rapidly at low concentrations of GA3 (up to 125 μM—circuit 3x, and 200 μM—circuit 1×). Then, any further increment became less evident with no significant statistical difference in the fluorescence levels at higher concentrations of GA3. Circuit 3× showed higher sensitivity to GA3 by returning an ON/OFF ratio equal to 2.46, whereas circuit 1× reached 2.18 only. The control circuit (in which the ScRNA was missing) did not return significant changes in fluorescence by varying GA3 concentration (see Supplementary Figure S4B and Supplementary Table S15).

We then used a different hormone as an input for a YES gate: the abscisic acid—ABA (37,39)—that drives the hetero-dimerization of ABI and PYL1 domains. At first, we fused the ABI domain to MCP and PYL1 to VP64 (see Supplementary Figure S4A-Design 2). This configuration, however, failed to respond to ABA (see Supplementary Figure S4C and Supplementary Table S17). We exchanged, then, ABI with PYL1, i.e. we fused the smaller domain PYL1 (183 AA) to MCP and the bigger domain ABI (301 AA) to VP64 (see Supplementary Figure S4A-Design 3). With this modification, ABA induced yEGFP expression considerably (see Figure 4B (also Supplementary Table S16)—and Supplementary Figure S4D (also Supplementary Table S18) for the control circuit) when the concentration ranged between 0 and 5 μM, at which the highest ON/OFF ratio was detected (a remarkable 8.21 in the 3× circuit, *P*-value < 0.0001, *n* = 3). For ABA concentrations over 5 μM, the fluorescence level first decreased slightly and then settled to a plateau.

After constructing the two YES gates successfully, we combined them into an AND gate for the simultaneous detection of ABA and GA3 (see Supplementary Figure S4E). We kept MCP fused to PYL1, whereas ABI was linked to GAI, and GID1 to VP64. As plotted in Figure 4C, the yEGFP expression level was significantly enhanced in the presence of both 5 μM ABA and 100 μM GA3 with respect to the ‘00′ state (i.e. in the absence of both chemicals) and the ‘01′ state (only GA3 is present). ABA alone, however, guaranteed a non-negligible activation (2.75-fold (*P*-value < 0.0001, *n* = 3, Supplementary Table S19) with respect to ‘00′), which is consistent with the rather high basal fluorescence in the GAI-GID1 system (Figure 4A). Overall, the AND gate reached a fairly good ON/OFF ratio equal to 2.44.

As a final comparison, we changed the activator design by fusing a hetero-dimerization domain directly to dCas12e and expressing an sgRNA targeting lexOpR (on the 3x circuit). We realized the ABA-sensing YES gate, first, by following our previous best implementation, i.e. by fusing PYL1 domain to dCas12e and VP64 to ABI. The ON/OFF ratio, at 5 μM ABI, was, however, a modest 1.50. By swapping the position of two hetero-dimerization domains (i.e. ABI was linked to dCas12e and PYL1 to VP64), the activation efficiency increased up to 2.79-fold (see Supplementary Figure S4F and G), still far from the 8.21-fold reached by dCas12e:ScRNA-L1. The GA-responding YES gate, where dCas12e was fused to GAI and VP64 to GID1, was unfunctional upon exposure to 100 μM GA3 (see Supplementary Figure S4H), which prevented the realization of a new version of the ABA-GA3 AND gate.

Taken together, the Boolean gates (YES and AND) here presented show that the dCas12e:ScRNA-L1 activator can be used as a component of chemically inducible systems. Moreover, the YES gates constructed by fusing an heterodimerization domain to dCas12e directly (which needed the expression of an unchanged sgRNA), turned out to be either less performant or even unfunctional. Hence, these results confirm that the scaffold RNA is an effective solution for genetic constructs.

### Two-gene regulation in a single circuit

**Figure 5.**
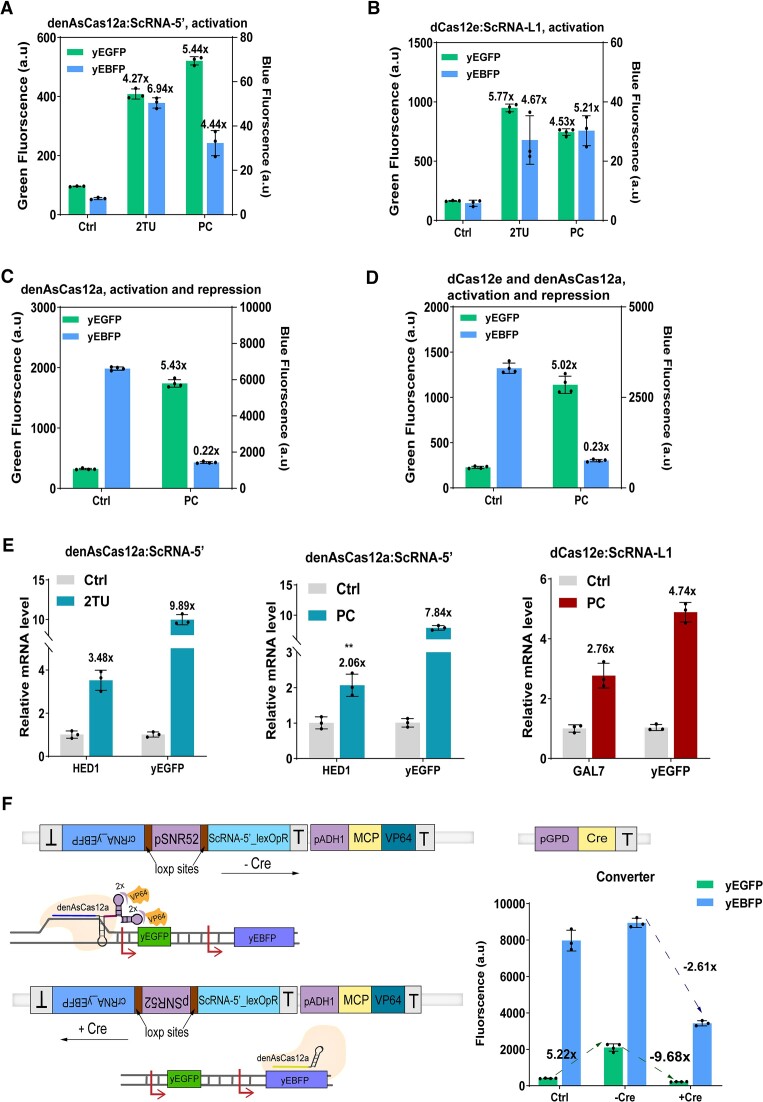
Two-gene transcription regulation. (**A**, **B**) denAsCas12a:ScRNA-5′ and dCas12e:ScRNA-L1 activate simultaneously the expression of yEGFP and yEBFP. (**C**) denAsCas12a activates yEGFP and represses yEBFP expression. (**D**) yEGFP is activated by dCas12e and yEBFP is repressed by denAsCas12a in the same circuit. (**E**) Activation of *S. cerevisiae* endogenous genes via dCas12:ScRNA. Three endogenous genes were selected: *HED1, GAL7* and *CYC1*. The native gene *ACT1* was used as internal reference for RT-qPCR measurements. ‘2TU’ means the ScRNAs are expressed via two different TUs. ‘PC’ refers to the ScRNAs that are expressed as components of a pre-crRNA. ‘Ctrl’ is the control circuit, where the ScRNA expression cassette is absent (see Supplementary Figure S5F). (**F**) Converter based on denAsCas12a. Recombinase (Cre) was put downstream of the constitutive *GPD* promoter. In the circuit without Cre expression, the ScRNA is synthesized and forms an activator with denAsCas12a, which triggers the production of yEGFP. The converter shift from yEGFP activation to yEBFP repression once Cre is produced. In the control circuit for the converter, Cre, crRNA, and ScRNA-5' are not expressed. Values on top of the bars refer to the ON/OFF ratio with respect to the control circuits. ‘**’*P*-value < 0.01; calculated via two-sided Welch's *t* test. Values near the dashed arrows represent the changes in relative fluorescence.

After showing that the complex made of a type V dCas12 protein and an ScRNA enhances the expression of a single gene considerably, we moved to the analysis of multiple gene activation. Initially, we placed *yEBFP* and *yEGFP* under the control of either denAsCas12a:ScRNA-5′ or dCas12e:ScRNA-L1 to check if both activators (generically referred to as dCas12:ScRNA) could activate two different genes simultaneously in the same circuit. *yEBFP* was under the same weak synthetic promoter used for yEGFP expression. Here, however, three tet operators (3xtetOp) replaced 3xlexOpR. We expressed each scaffold RNA in two distinct ways (see Supplementary Figure S5A and B). One required two transcription units (hence, it was termed 2TU) to produce the two ScRNAs (targeting tetOp and lexOpR) separately. The other way made use of a single transcription unit where the two ScRNAs were organized into a short pre-crRNA (PC). Here, the two ScRNAs were separated either by the DR of denAsCas12a—which was processed by the same denAsCas12a—or by an endogenous tRNA^Gly^ cleaved by the RNase P and RNase Z present in the *S. cerevisiae* cells (3,40–42). The results in Figure 5A and B show that the two configurations worked well both with denAsCas12a and dCas12e by causing a significant activation (over 4-fold, see Supplementary Table S20–S21) of yEGFP and yEBFP. It should be noted that dCas12e displayed less than half of the activation efficiency on a single target (5.77-fold instead of 13.02-fold), which might indicate that the activity of a dCas12e:ScRNA-L1 activator is dependent on the relative amount of MCP-VP64.

Next, we constructed two circuits that contained both activation (yEGFP) and repression (yEBFP) of transcription via denAsCas12a either alone (see Supplementary Figure S5C) or together with dCas12e (see Supplementary Figure S5D). As discussed above, dCas12e can be used as an activator only. The *yEBFP* gene was put downstream of the strong *GPD* promoter to detect more easily a drop in blue fluorescence. Both circuits made use of a pre-crRNA hosting a scaffold RNA to activate *yEGFP* expression (by targeting lexOpR) and a denAsCas12a crRNA to repress blue fluorescence (by binding a portion of the *yEBFP* gene). Each circuit utilized the endoribonuclease activity of denAsCas12a to process the pre-crRNA and separate the scaffold RNA, used in activation, from the crRNA employed in repression. Results in Figure 5C and Supplementary Table S22 show that denAsCas12a alone is sufficient to achieve a strong activation of *yEGFP* (5.43-fold) and a remarkable repression of *yEBFP* (0.22-fold). Figure 5D (also Supplementary Table S23) points out that also dCas12e:ScRNA-L1 activates *yEGFP* considerably (5.02-fold) without interfering with *yEBFP* inhibition (0.23-fold) by denAsCas12a.

After showing that dCas12:ScRNA triggered the expression of (multiple) exogenous genes (*yEGFP* and *yEBFP*), we have checked how these new activators performed on endogenous genes. We applied denAsCas12a:ScRNA-5′ to *HED1* and *GAL7* (29) and dCas12e:ScRNA-L1 to *GAL7* and *CYC1* (30). The choice of the endogenous genes was conditioned by the PAM of the two Cas proteins, that are slightly different. To facilitate the screening of the strains engineered for endogenous gene regulation, we expressed an ScRNA sequence for yEGFP activation together with the ScRNA designed for the endogenous gene. Since the two ScRNAs were always placed on the same plasmid, a high fluorescence level indicated a successful integration of both scaffold RNAs. Therefore, the strains were selected via FACS experiments and then endogenous gene expression was quantified by carrying out RT-qPCR. Initially, we used expression cassettes consisting of two different TUs to generate the ScRNAs (see Supplementary Figure S5E and F). Each activator enhanced the endogenous gene expression above its basal level in a statistically significant way. The activation efficiency was not as high as that measured on the exogenous genes, probably because the latter had three lexOpR target sites, whereas the former only one. The activation of *HED1* (by denAsCas12a:ScRNA-5′) showed the highest ON/OFF ratio: 3.48 (*P*-value = 0.0035, *n* = 3, see Supplementary Table S24), whereas that of *GAL7* and *CYC1*—due to the action of dCas12e:ScRNA-L1—was less than two (see Figure 5E and Supplementary Figure S5F, and Supplementary Table S25).

For the next measurements, we made use of a single-unit expression system where the two scaffold RNAs were arranged in a pre-crRNA and separated by either the denAsCas12a direct repeat or a tRNA^Gly^ sequence. dCas12e:ScRNA-L1 returned the highest activation for *GAL7* (ON/OFF ratio: 2.76, *P*-value = 0.0080, n = 3), whereas denAsCas12a:ScRNA-5′ was only moderately performant on *HED1* and *GAL7* (see Figure 5E and Supplementary Figure S5F).

To exclude off-target effects, transcriptome analysis was carried out. Results showed that several genes were over- and under-expressed, especially in the presence of dCas12e:ScRNA-L1. Since a computational investigation on the whole *S. cerevisiae* genome excluded the presence of lexOpR sequences preceded by TTCN (and TTTV), we think that genes underwent up- or down-regulation due to stress conditions of the engineered cells (see Supplementary Tables S32–S35 and Supplementary Figure S8).

In short, dCas12:ScRNA-based activators enhanced rather moderately the expression of endogenous genes. However, exogenous genes were highly activated when three copies of the target sequence (either lexOpR or tetOp) were present. Therefore, the activation efficiency of dCas12:ScRNA on endogenous genes might be improved by expressing more ScRNAs that target different locations of the promoter upstream of the selected genes. This is, however, possible only if multiple Cas12 PAMs are available in front of the core promoter region. It should be also noted, though, that transcription activation does not depend on the sole number of activator binding sites but also on their reciprocal distance and the spacing between them and the TATA box(es) (43). Hence, ScRNA spacer selection and design is a crucial task to maximize the activation of endogenous genes in *S. cerevisiae*.

Finally, we built a more complex system, i.e. a converter ‘activation to repression’ circuit by controlling, through a FLEx switch, the direction of the *SNR52* promoter (pSNR52) in charge of synthesizing denAsCas12a ScRNA (for activation) or crRNA (for repression) (44,45). As shown in Figure 5F and Supplementary Table S26, the scaffold RNA (targeting lexOpR) is transcribed in the absence of recombinase (- Cre) and leads to an evident increase in green fluorescence (5.22-fold, *P*-value = 0.0005, *n* = 4) with respect to the control circuit where, besides Cre, both ScRNA and crRNA are not expressed. In the presence of Cre (+ Cre), the direction of pSNR52 is reversed, which results in the synthesis of denAsCas12a crRNA (targeting *yEBFP*) that causes a decrease in yEBFP expression (2.61-fold, *P*-value < 0.0001, *n* = 3). Moreover, without the ScRNA binding lexOpR, yEGFP can no longer be produced and a drop in green fluorescence occurs too. A similar behavior was detected from the ‘Converter-Met’ circuit, where the *cre* gene lies downstream of the *MET25* regulated promoter (pMET25), which is repressed by methionine (Met) (26,46) (see Supplementary Figure S5G and Supplementary Table S27).

Overall, our results indicate that both dCas12e and denAsCas12a are able to activate, separately, two different genes (both exogenous and endogenous) effectively upon expression of two scaffold RNAs that differ only for the spacer sequence. Moreover, denAsCas12a can carry out activation and repression within the same circuit if an ScRNA (activation) and a crRNA (repression) are designed properly. This further illustrates that the engineering of a guide RNA permits to achieve different functions inside the cells in a relatively easy and quick way.

### Regulation of multiplex genes and β-carotene pathway via dCas12:ScRNA

**Figure 6.**
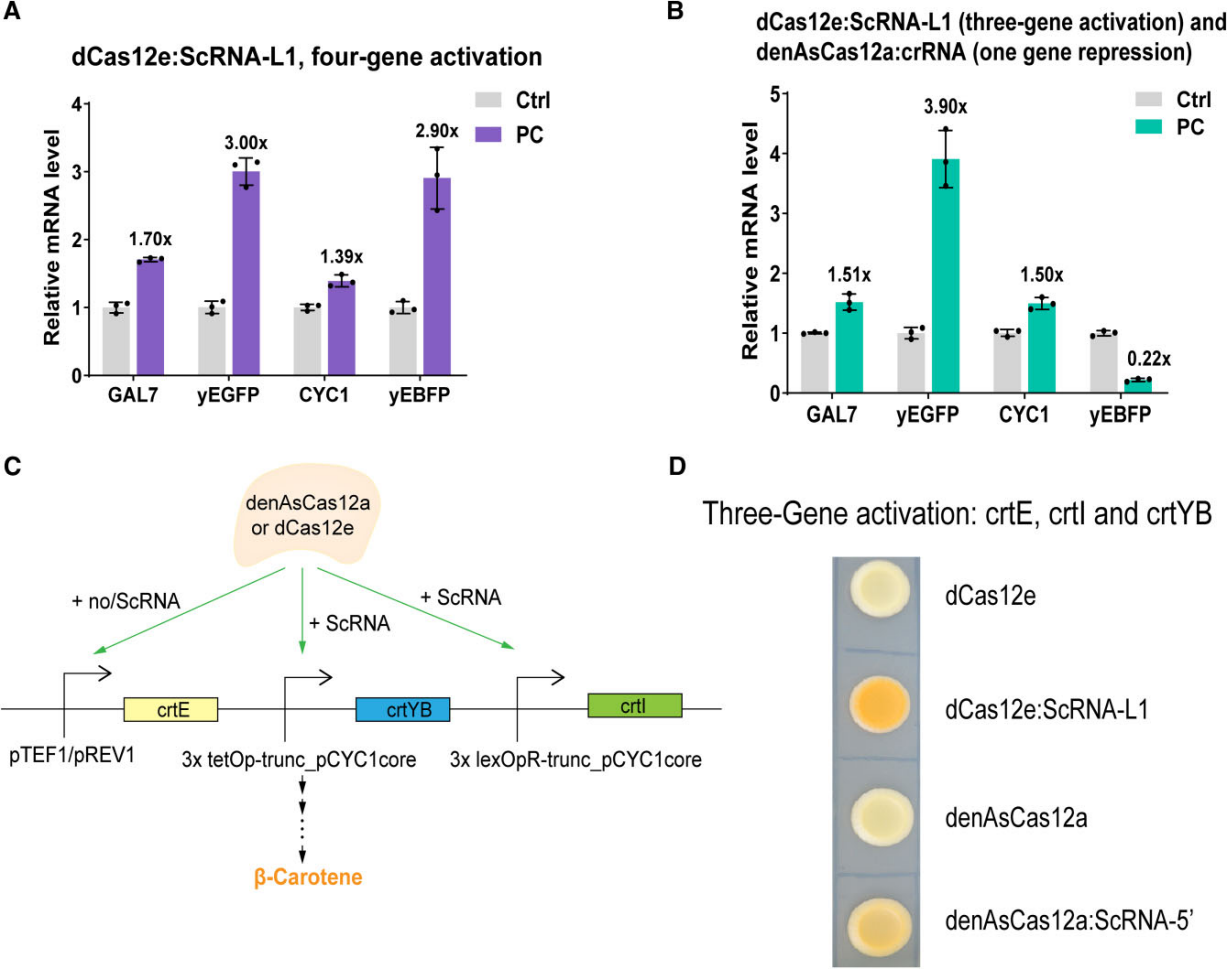
Multigene regulation based on dCas12:ScRNA. (**A**) dCas12e:ScRNA-L1 activated four target genes successfully. (**B**) Beside the activation of *GAL7*, *yEGFP*, and *CYC1* by dCas12e:ScRNA-L1, *yEBFP* transcription is strongly inhibited by denAsCas12a:crRNA. (**C**) A synthetic β-carotene metabolic pathway, where the expression of either two (*crtYB* and *crtI*) or all three genes is enhanced by dCas12:ScRNA activators. (**D**) β-carotene production via three-gene activation. The selected yeast strains grew for 6 days at 30°C. Each activated pathway confers to the cells some orange coloration, whereas the basal β-carotene expression (i.e. in the absence of any ScRNAs) is almost undetectable. The best result obtained with a three-gene activation was from dCas12e:ScRNA-L1 (see also Supplementary Figure S7B).

We continued the analysis of multiple-gene-expression control by dCas12:ScRNA by building new, longer pre-crRNA sequences, which allowed regulating the transcription of up to four genes, both endogenous and exogenous. We targeted the same genes used in the previous experiments, namely: *HED1*, *GAL7, CYC1*, and the two genes encoding for the fluorescent proteins (*yEGFP* and *yEBFP*). We constructed two transcription units to produce four ScRNAs for dCas12e- and denAsCas12a-mediated activation (see Supplementary Figure S6A and B). dCas12e activated the expression of all four genes (*GAL7*, *yEGFP*, *CYC1*, and *yEBFP*), even though the ON/OFF ratios were lower than those measured on two genes only, ranging from 1.39 (*CYC1*) to 3.00 (yEGFP) (see Figure 6A and Supplementary Table S28). This result seems to confirm that the activation efficiency of dCas12e:ScRNA-L1 depends considerably on the amount of MCP-VP64 in the cell. In contrast, denAsCas12a showed a clear preference for the two foreign genes (*yEGFP* and *yEBFP*) with an activation efficiency above 7-fold on each of them. While *GAL7* activation was comparable to that obtained in the two-gene activation circuits, *HED1* resulted even inhibited as if MCP-VP64 failed to bind the scaffold RNA targeting pHED1, turning, in this way, denAsCas12a:ScRNA-5′ into a repressor (Supplementary Figure S6B and Supplementary Table S30).

As a next step, we used dCas12 to carry out both activation and repression on four genes. dCas12e, however, cannot work as a repressor and, for this task, required the presence of denAsCas12a. In both circuits for simultaneous gene activation and repression, the cassette for the pre-crRNA synthesis contained three ScRNAs (either ScRNA-5′ or ScRNA-L1, for activation) and one denAsCas12a crRNA (to repress only *yEBFP*—see Supplementary Figure S6C and D). As shown in Figure 6B and Supplementary Table S29, dCas12e:ScRNA-L1 activated moderately *GAL7* and *CYC1*, and more strongly *yEGFP*. Moreover, denAsCas12a:crRNA reduced yEBFP expression drastically (78%). In contrast, denAsCas12a:ScRNA-5′ activated *yEGFP* (highly) and *GAL7* (modestly), whereas *HED1* activation failed again. *yEBFP* was correctly inhibited, though only of 21% according to the mRNA expression level (see Supplementary Figure S6D and Supplementary Table S31).

On the whole, dCas12e:ScRNA-L1 (alone or together with denAsCas12a:crRNA) was able to regulate up to four genes in a single circuit. denAsCas12a:ScRNA in contrast, failed twice—when targeting four genes—to activate *HED1*, whereas the same gene was well-activated inside two-gene-target circuits.

As a further test, we applied the dCas12:ScRNA activators to the production of β-carotene. The β-carotene pathway was engineered by integrating three genes (*crtE*, *crtYB*, and *crtI* (47)) in the *S. cerevisiae* genome. Initially, we activated the expression of two genes only: *crtYB* (placed downstream of 3xtetOp-truncated_pCYC1core) and *crtI* (3xlexOpR-truncated_pCYC1core), whereas crtE was constitutively produced under the *TEF1* promoter (pTEF1). Then, we controlled the synthesis of the three proteins by replacing pTEF1 with the much weaker *REV1* constitutive promoter (pREV1, see Supplementary Table S1) whose transcription initiation rate was enhanced by the action of dCas12:ScRNAs directly (see Figure 6C). The qualitative results from the integration plates (see Figure 6D and Supplementary Figure S7B) appeared to confirm the higher efficiency of dCas12e:ScRNA-L1 in the simultaneous activation of more than two genes.

## Discussion

In this work, we have shown that dCas12e, whose molecular size is much smaller (∼120 KDa) than that of dCas9 and dCas12a (∼160 KDa) (14,48,49), is a useful template for activator engineering. Differently from other type V CRISPR-associated proteins, dCas12e has no RNase functionality. However, multiplex regulation, via the expression of a pre-crRNA, is possible by joining different sgRNAs via tRNA^Gly^ that are cleaved by RNase P and Z, already present in yeast cells.

dCas12a has been used, since its discovery, to regulate gene expression in different organisms. However, so far, new transcription factors have been constructed by modifying dCas12a directly, e.g. through the fusion with activation or repression domains. In this work, we have introduced a new way to turn dCas12a (and dCas12e) into TFs in *S. cerevisiae* cells. Our method demands to modify a mature crRNA (sgRNA) with the insertion of hairpins that permit to recruit activation domains linked to RNA-binding motifs. In other words, we have shown that, beside CRISPR-Cas9, a scaffold RNA (ScRNA) can be engineered also for type V CRISPR-Cas12 systems. Our results underline the fact that a scaffold RNA can be engineered for CRISPR-Cas systems that rely either on a crRNA (e.g. CRISPR-denAsCas12a) or on a single guide RNA (such as CRISPR-Cas12e), where the crRNA is fused to a portion of the tracrRNA (which, in general, provides more positions for the hairpin insertion).

We have constructed a variety of ScRNAs for both dCas12e and denAsCas12a. As for dCas12e, we have tested five elements in ScRNA engineering: (i) four different RNA hairpin—coat protein pairs: MS2-MCP, PP7-PCP, Qβ-QCP and com-Com, (ii) single or double RNA hairpin, (iii) three possible positions (5′-end, loop 1 and loop 2) in the sgRNA, (iv) the usage of the bare dCas12e or the chimera dCas12e-VPR, (v) the quantity of VP64. Based on the results we obtained in the construction of dCas12e ScRNA, in order to engineer denAsCas12a ScRNA we have considered only three variables, namely: (i) the crRNA regions to modify (only two: 3′-end and 5′-end), (ii) mutated DRs and (iii) the length of the linker between the RNA hairpin and the mDR. The optimal ScRNA configurations were obtained by inserting 2× MS2 into the loop 1 region of dCas12e sgRNA and the 5′-end of denAsCas12a crRNA (here, together with a of 5- or 9-bp-long linker to what we termed mDR1). We refer to these two configurations as ScRNA-L1 and ScRNA-5′, respectively. Our results showed that the activators based on ScRNA guaranteed higher performance than that of the activators made by modifying dCas12 directly, which is similar to dCas9 scenario (16,18,50).

As possible first applications, we developed Boolean gates (YES and AND) where the dCas12e:ScRNA-L1 activator functionality was triggered by the plant hormones GA3 and ABA. Boolean (logic) circuits are important artifacts in Synthetic Biology and CRISPR-Cas systems have been already exploited as components of novel sensing devices for medical diagnostics (11,26,37). Moreover, Truong *et al.* developed a gene therapy method by utilizing a split Cas9 system (51), whereas Nihongaki *et al.* established an inducible platform for genome editing and gene regulation based on the CRISPR-Cas12a system (52). Our circuits that fuse aptamers bound by inducible hetero-domains to type V sgRNAs present a novel solution for the assembly of type V CRISPR-Cas-based logic gates.

Besides digital circuits, we assembled a synthetic converter that switches from activation to repression by controlling denAsCas12a:ScRNA-5′ expression.

We proved that dCas12e:ScRNA-L1 and denAsCas12a:ScRNA-5′ activate foreign genes strongly and endogenous genes more moderately. However, we think that the expression level of the latter could be increased just by expressing more ScRNAs targeting the promoter upstream of the selected genes. dCas12e:ScRNA-L1 is capable of multigene (up to four) activation, whereas denAsCas12a:ScRNA-5′ worked reliably only on two-gene systems. The results about β-carotene further confirmed that dCas12e:ScRNA is more reliable in multigene activation with respect to denAsCas12a:ScRNA-5′. However, differently from dCas12e, denAsCas12a can carry out transcriptional repression. Cas12e failure in down-regulating gene expression seems to point out that dCas12e:sgRNA binds a target DNA sequence more weakly than d(Lb/enAs)Cas12a:crRNA or dSpCas9:sgRNA. This disparity in DNA affinity could also explain the lower activation due to dCas12e-containing ribonucleoproteins than that achieved by activators built on different dCas:crRNA/sgRNA systems.

Taken together, the establishment of ScRNA in type V CRISPR-Cas system not only improves the activation efficiency of dCas12-based activators but also broaden the design of Synthetic Biology circuits and biotech systems.

## Supplementary Material

gkad1216_Supplemental_Files

## Data Availability

All FCS files measured by FACS machine are available in the FlowRepository under accession IDs FR-FCM-Z69U (dCas12:ScRNA-2) and FR-FCM-Z6RM (dCas12:ScRNA-3). Further data is available in the Gene Expression Omnibus at https://www.ncbi.nlm.nih.gov/geo/ under accession code GSE249355.
