# Supplementary material for: Scaffold RNA engineering in type V CRISPR-Cas systems: a potent way to enhance gene expression in the yeast Saccharomyces cerevisiae
Source: Nucleic Acids Res. 2023 Dec 24;52(3):1483–97. doi: 10.1093/nar/gkad1216 (PMC10853767; doi:10.1093/nar/gkad1216)
Supplement: gkad1216_Supplemental_Files [file gkad1216_supplemental_files.zip › NAR-MAM-YLF-SM-rev.pdf]

Supplementary Materials

Scaffold RNA engineering in type V CRISPR-Cas systems: a potent way to enhance gene expression in the yeast *Saccharomyces cerevisiae*.

Lifang Yu[1], and Mario Andrea Marchisio[1*]

[1] School of Pharmaceutical Science and Technology, Tianjin University, 92 Weijin Road, 300072-Tianjin, China

*Correspondence: mario@tju.edu.cn or mamarchisio@yahoo.com

1. Supplementary Figures

A

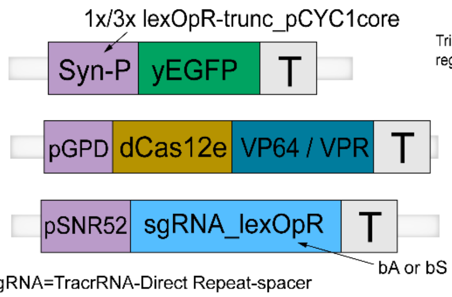

B

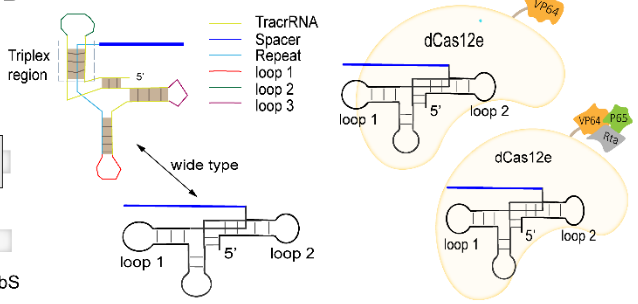

C

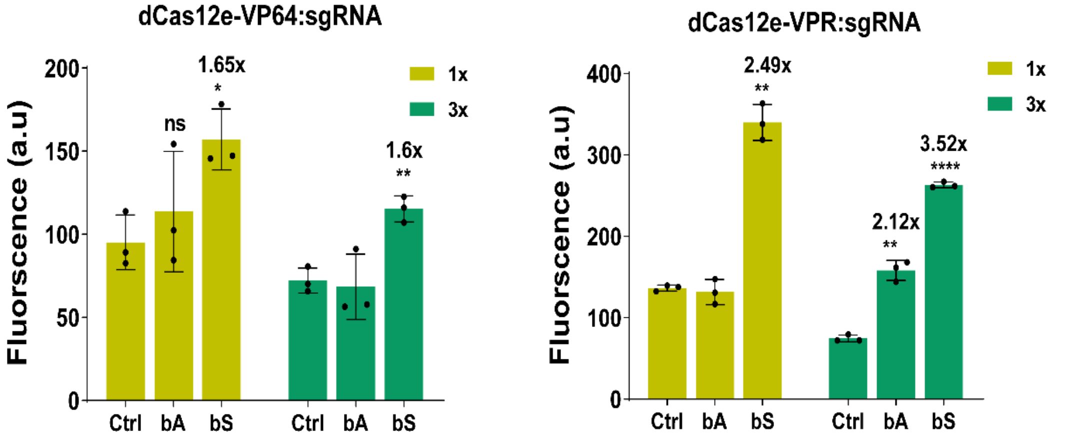

1x (ON/OFF)-bA	denAsCas12a	dLbCas12a	dCas12e	1x (ON/OFF)-bS	denAsCas12a	dLbCas12a	dCas12e
VP64	1.84	ns	ns	VP64	1.45	ns	1.65
VPR	2.62	2.03	ns	VPR	2.3	2.31	2.49
3x (ON/OFF)-bA	denAsCas12a	dLbCas12a	dCas12e	3x (ON/OFF)-bS	denAsCas12a	dLbCas12a	dCas12e
VP64	2.78	2.34	ns	VP64	2.01	1.63	1.6
VPR	2.93	3.21	2.12	VPR	4.45	2.77	3.52

D

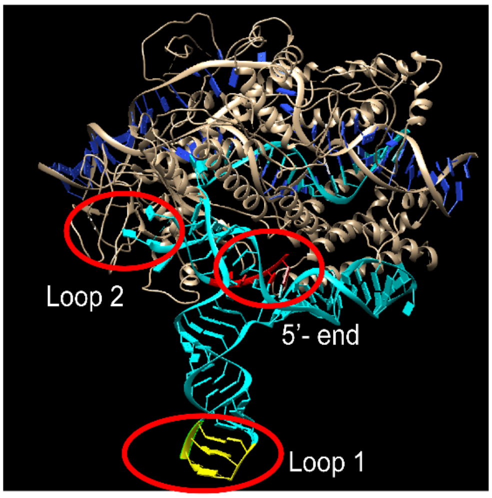

E

Name	1x	3x	(3x)/(1x)
MS2	3.66	6.1	1.67
PP7	3.28	5.7	1.74
Qβ	2.17	4.5	2.07
com	1.58	2.66	1.68
1x	1 hairpin	2 hairpins	2 hairpins/1 hairpin
MS2	3.66	4.05	1.11
PP7	3.28	5.83	1.78
3x	1 hairpin	2 hairpins	2 hairpins/1hairpin
MS2	6.1	13.02	2.13
PP7	5.7	6.4	1.12

Figure S1. The activation efficiency of dCas12e-AD:sgRNA and dCas12e:ScRNA. (**A**) The circuits containing dCas12e-AD:sgRNA. The activation domain (VP64 or VPR) was fused to dCas12e to generate a chimeric protein: dCas12e-VP64 or dCas12e-VPR. The chimeric protein forms a complex with the sgRNA—composed of the tracrRNA, the direct repeat, and the spacer binding the short lexOpR sequence)—and activates the expression of yEGFP (from a synthetic promoter, Syn-P, containing 1 or 3 copies of lexOpR (1)). 'bA' represents an sgRNA that binds the anti-sense strand of the target DNA, 'bS' refers to an sgRNA binding the sense strand. (**B**) Graphical representation of the dCas12e-AD:sgRNA complex. (**C**) The activation efficiency of dCas12e-VP64/VPR:sgRNA on a single or a triple lexOpR. 'Ctrl' means the control circuit in which the sgRNA-expressing transcription unit is missing. The left panel shows the activation due to dCas12e-VP64:sgRNA, whereas the right panel refers to the action of dCas12e-VPR:sgRNA. Values above the lines are the activation folds with respect to the 'Ctrl' group. Star symbols (*) correspond to different p-values that were calculated via two-sided Welch's *t* test: '*', p-value <0.05; '**', p-value <0.01; '****'; p-value <0.0001; 'ns', no significant statistical difference. The table under the bar plots compares the activation efficiency of three dCas12-based activators. Data used in this table about denAsCas12a and dLbCas12a are obtained from (1). (**D**) The 3D structure of the Cas12e:sgRNA:DNA complex (see (2) and PDB: 6ny2). The golden part represents Cas12e, the blue one is the target DNA, and the cyan one refers to the sgRNA. The three sgRNA locations, termed loop 1, loop 2, and 5'-end, are tagged with a red circle. (**E**) The activation efficiency due to RNA hairpin—coat protein pairs interacting with the ScRNA containing a single or a double MS2 hairpin on loop 1.

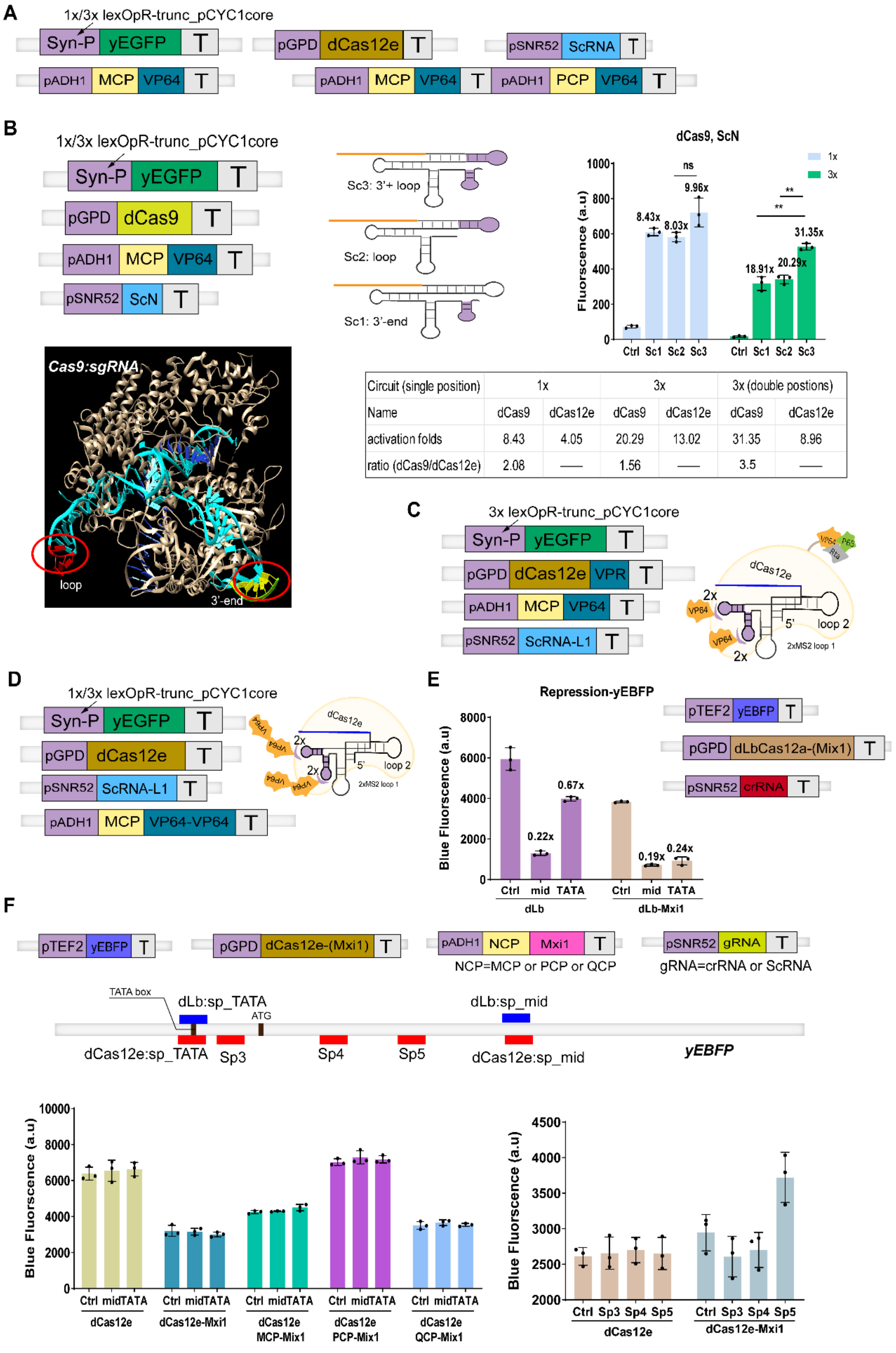

Circuit (single position)	1x		3x		3x (double postions)	
Name	dCas9	dCas12e	dCas9	dCas12e	dCas9	dCas12e
activation folds	8.43	4.05	20.29	13.02	31.35	8.96
ratio (dCas9/dCas12e)	2.08	——	1.56	——	3.5	——

Figure S2. (**A**) The transcription units in circuits expressing the ScRNAs in Figure 2A. (**B**) dCas9:ScRNA activators: TUs, ScRNA design, 3D Cas9:sgRNA structure, activation efficiency, and comparison with dCas12e:ScRNA-L1. Three ScRNAs were constructed

by using the 2xMS2 motif. Sc1 was built by inserting 2xMS2 on the 3'-end of Cas9 sgRNA. Sc2 was made by placing 2xMS2 in the section (the loop) joining the tracrRNA and the crRNA repeat. Sc3 merged the architectures of Sc1 and Sc2. 'ScN' means any among Sc1, Sc2, and Sc3. The structure of dCas9:sgRNA was obtained from (3). Sc3 shows higher performance than Sc1 and Sc2 only in the 3x circuit. '*': p-value < 0.05; '**': p-value <0.01; 'ns', no significant statistical difference (calculated via two-sided Welch's *t* test). (**C**, **D**) Transcription units of the circuits characterized by the presence of dCas12e-VPR and MCP-2xVP64, respectively. The ScRNA hosts a double MS2 hairpin on loop 1. (**E**) TUs to express dLbCas12a-based repressors and the repressor effects on blue fluorescence. 'Ctrl' are circuits without crRNA. Values on the top of bars refer to repression efficiency with respect to the 'Ctrl' circuits; 'mid' and 'TATA' positions are shown in (F) as blue rectangles. (**F**) TUs used to test either dCas12e(-Mxi1):sgRNA or dCas12e:ScRNA together with MCP/PCP/QCP-Mxi1 as repressors. Diagram showing the locations (red rectangles), along the *yEBFP* gene, targeted by dCas12e-based repressors. Blue fluorescence levels of the complete and control circuits (where either the sgRNA or the ScRNA is missing).

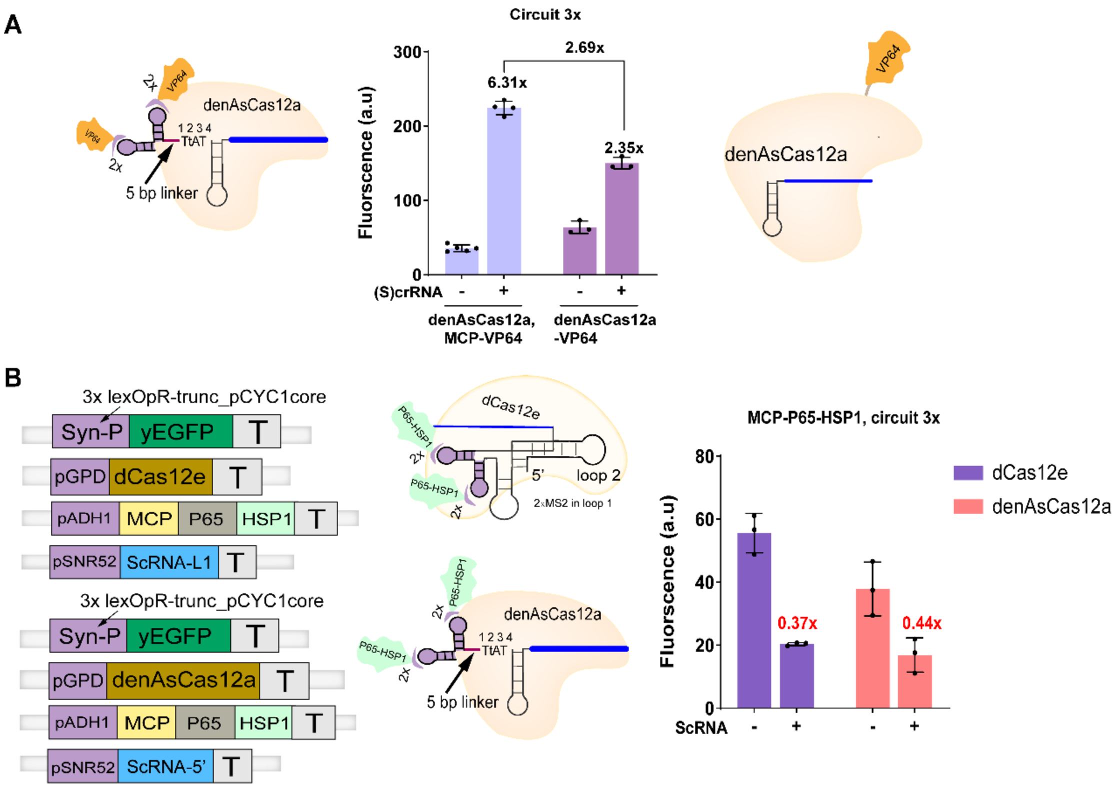

Figure S3. (**A**) Comparison of the activation efficiency of denAsCas12a:ScRNA-5’ and denAsCas12a-VP64:crRNA in the 3x circuit. denAsCas12a:ScRNA-5’ is 2.69-fold more efficient than denAsCas12a-VP64:crRNA in enhancing the green fluorescence expression level. (**B**) Coupling the MCP-P65-HSP1 activation domain to dCas12:ScRNA in *S. cerevisiae*. From the left: the two circuits involving MCP-P65-HSP1 AD; schematics of the new ribonucleoproteins; activation efficiency in the 3x circuit. The bar plot shows that the ScRNAs together with the MCP-P65-HSP1 AD performed repression rather than activation of the green fluorescence signal. Values on top of the “+” (complete circuit) bars are the “activation” folds with respect to the to control group (“-”), where the ScRNA/crRNA is not expressed.

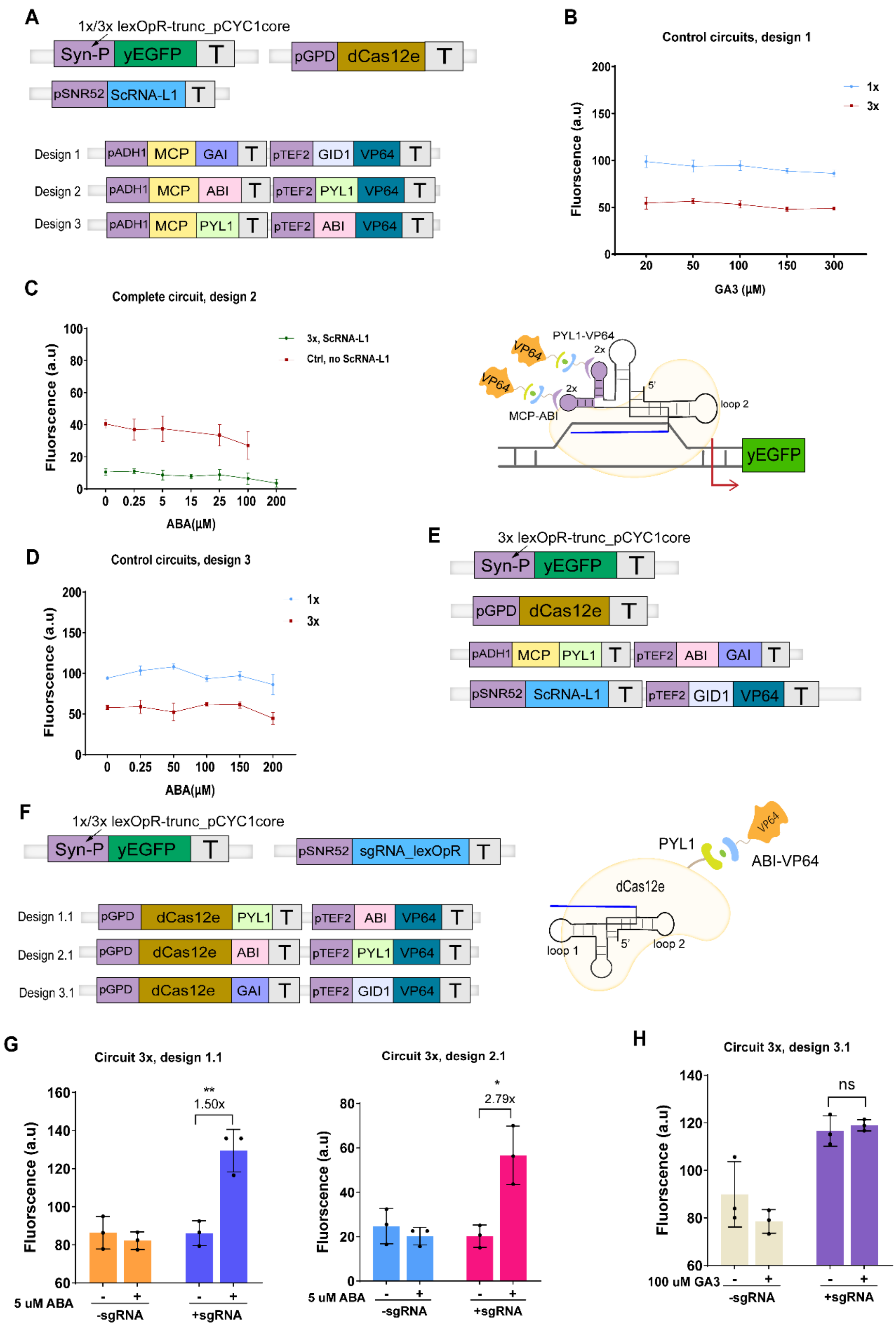

Figure S4. Construction of plant-hormone inducible systems. (**A**) The sketch of three inducible circuits. The hetero-dimerization domains are fused to MCP and VP64. Their expression cassettes are placed together on a single vector. (**B**) Control circuit, Design

1: GA3 titration curve. (**C**) Full circuit, Design 2: ABA titration curve. Fluorescence expression does not increase with higher concentrations of ABA. (**D**) Control circuit, Design 3: ABA titration curve. (**E**) The transcription units used in the AND gate construction. The AND gate scheme follows Design 3 where VP64 is replaced by GAI, and the fusion protein GID1-VP64 is added. (**F**) Diagrams of the three circuits employing an sgRNA instead of ScRNA-L1. One dimerization domain is fused to dCas12e, the other to VP64 to form an activator in the presence of the plant hormone, as depicted in the right panel. (**G**) and (**H**) Activation efficiency of the systems in (F). "-sgRNA" refers to the circuits without sgRNA synthesis; "+sgRNA" means circuits containing sgRNA expression cassette (complete circuits). Their numerical value (fold-change) is written (when available) in each bar plot. '*': p-value <0.05; '**': p-value <0.01; 'ns', no significant statistical difference (calculated via two-sided Welch's *t* test). Every control circuit lacks the ScRNA-L1/sgRNA.

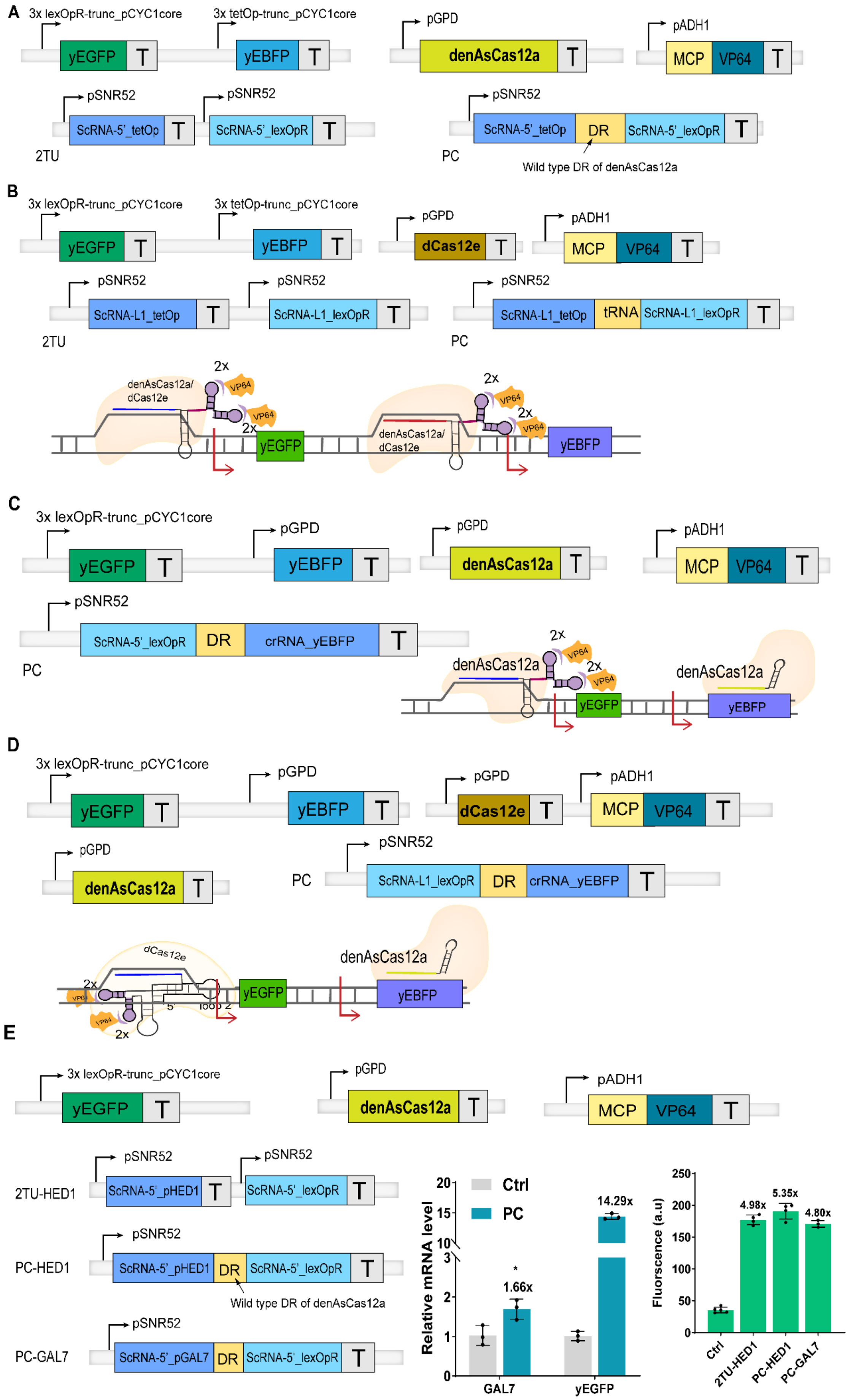

A
3x lexOpR-trunc_pCYC1core
3x tetOp-trunc_pCYC1core
pGPD
pADH1
yEGFP
T
yEBFP
denAsCas12a
MCP
VP64
pSNR52
ScRNA-5'_tetOp
ScRNA-5'_lexOpR
2TU
PC
DR
Wild type DR of denAsCas12a
B
dCas12e
ScRNA-L1_tetOp
ScRNA-L1_lexOpR
tRNA
2x
denAsCas12a/
dCas12e
C
crRNA_yEBFP
denAsCas12a
D
E
2TU-HED1
PC-HED1
PC-GAL7
ScRNA-5'_pHED1
ScRNA-5'_pGAL7
Ctrl
PC
Relative mRNA level
GAL7
yEGFP
1.66x
14.29x
Fluorscence (a.u)
4.98x
5.35x
4.80x

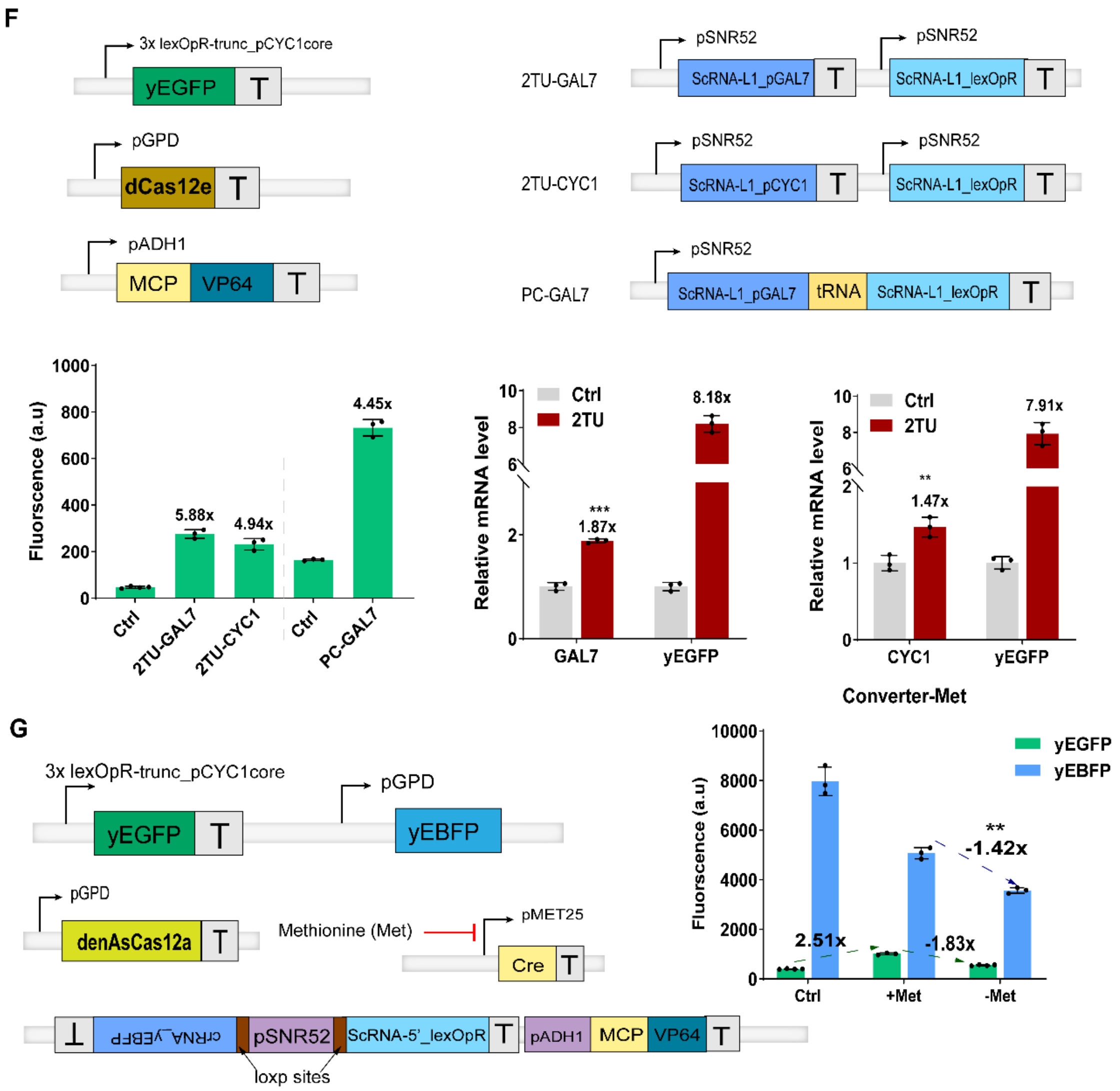

Figure S5. The transcription units used in the circuits involving two-gene transcription regulation. (**A, B**) Simultaneous activation of both *yEGFP* and *yEBFP* genes by either denAsCas12a- or dCas12e-based activators. *yEBFP* is placed downstream of the weak “trunc_pCYC1core” preceded by three tet operators (3xtetOp) (i.e., 3xtetOp-trunc_pCYC1core). The ScRNAs are expressed in two different ways. One consists of two separate transcription units (2TU), the other requires a single transcription unit for the synthesis of a short pre-crRNA (PC). On the pre-crRNA, the two ScRNAs are separated by either denAsCas12a DR or a tRNA^{Gly} sequence. (**C, D**) denAsCas12a alone or together with dCas12e realizes the simultaneous activation of yEGFP and repression of yEBFP. (**E**) Circuits to enhance the expression of an endogenous gene (*HED1* or *GAL7*) together with *yEGFP* via denAsCas12a:ScRNA-5’. Activation efficiency is determined via RT-qPCR (*HED1* results are shown in Figure 5E) and fluorescence measurement (FACS). ‘Ctrl’ refers to circuits without the ScRNA-5’ expression cassette. (**F**) Circuits to enhance the expression of a single endogenous gene (*CYC1* or *GAL7*) together with *yEGFP* via dCas12e:ScRNA-L1. Activation efficiency is measured via RT-qPCR (see also Figure 5E) and fluorescence detection (FACS). ‘Ctrl’ represents

circuits where ScRNA-L1 is not expressed. In (E, F), the values on the top of bars are the ON/OFF ratios with respect to the 'Ctrl' group. (**G**) The transcription units used in the 'Converter-Met' construction. Loxp sites (loxp-loxp2272) (4), arranged in opposite directions, are separated by pSNR52. The expression of Cre enzyme is controlled by pMET25—repressed in the presence of 10 mM methionine (5). Engineered cells, which grew in a synthetic medium supplied with 10 mM Met, showed an increase in yEGFP expression (2.51-fold) with respect to the control circuit that lacks Cre, ScRNA-5', and crRNA. In the absence of Met, Cre is expressed, which provokes a small decrease both in blue and green fluorescence signal (less than two-fold). The low performance of the 'Converter-Met' circuit is due to a non-negligible leakage of pMET25, which causes a decrease in blue fluorescence also in the presence of Met (+Met). Values near the dashed arrows represent the changes in relative fluorescence. '*': p-value <0.05; '**': p-value <0.01; '***': p-value <0.001; 'ns': no significant difference (calculated via two-sided Welch's *t* test)

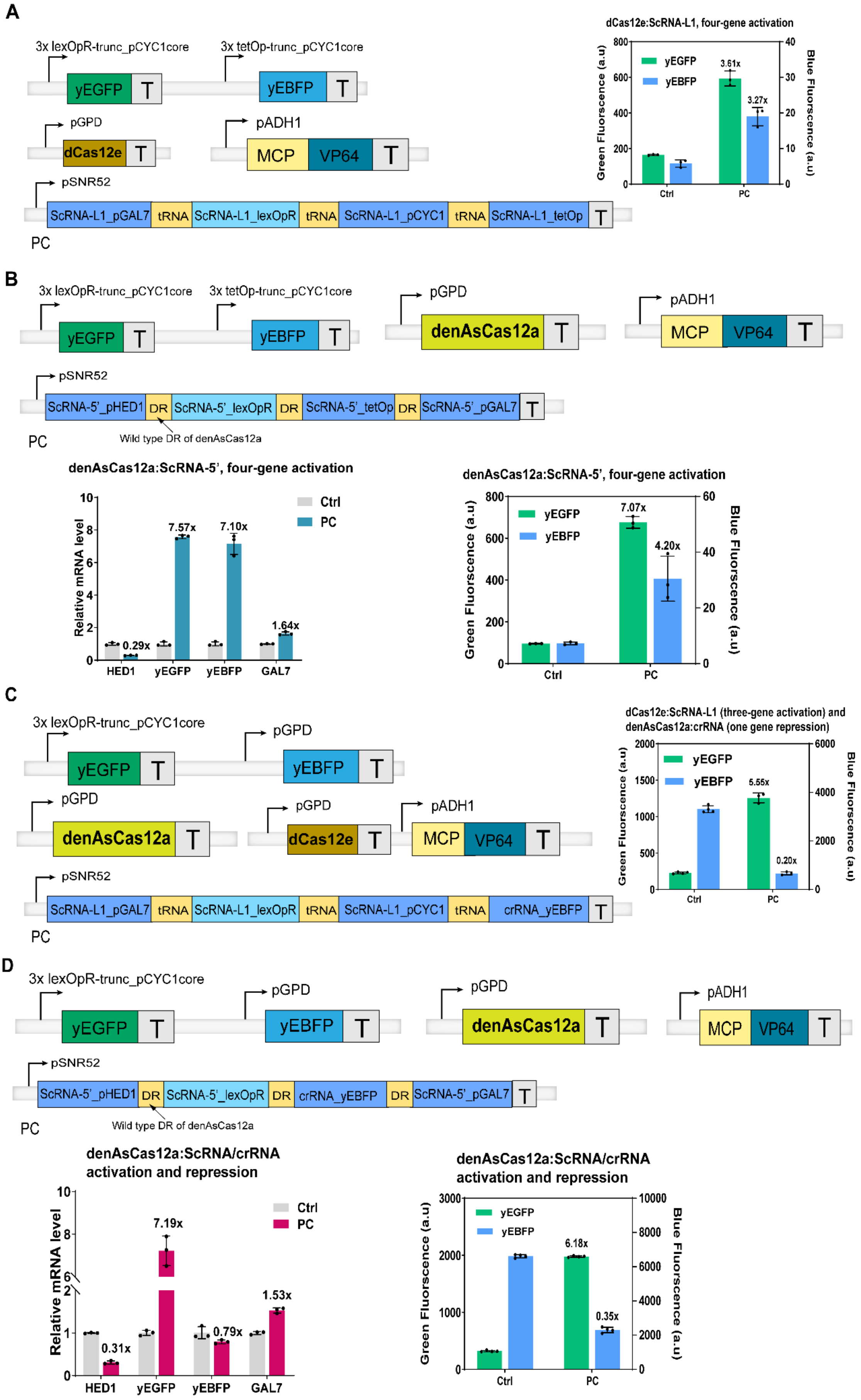
A
3x lexOpR-trunc_pCYC1core
3x tetOp-trunc_pCYC1core
yEGFP
T
yEBFP
pGPD
dCas12e
pADH1
MCP
VP64
pSNR52
ScRNA-L1_pGAL7
tRNA
ScRNA-L1_lexOpR
ScRNA-L1_pCYC1
ScRNA-L1_tetOp
PC
dCas12e:ScRNA-L1, four-gene activation
Green Fluorescence (a.u)
Blue Fluorscence (a.u)
3.61x
3.27x
Ctrl
B
denAsCas12a
ScRNA-5'_pHED1
DR
ScRNA-5'_lexOpR
ScRNA-5'_tetOp
ScRNA-5'_pGAL7
Wild type DR of denAsCas12a
denAsCas12a:ScRNA-5', four-gene activation
Relative mRNA level
0.29x
7.57x
7.10x
1.64x
HED1
GAL7
Green Fluorscence (a.u)
Blue Fluorscence (a.u)
7.07x
4.20x
C
crRNA_yEBFP
dCas12e:ScRNA-L1 (three-gene activation) and
denAsCas12a:crRNA (one gene repression)
5.55x
0.20x
D
denAsCas12a:ScRNA/crRNA
activation and repression
0.31x
7.19x
0.79x
1.53x
Green Fluorescence (a.u)
Blue Fluorescence (a.u)
6.18x
0.35x

Figure S6. Multiple-gene transcription regulation by dCas12:ScRNAs. (**A**,**B**) Four-gene activation. Transcription units and results from RT-qPCR (*ACT1* was used as an internal reference gene) and fluorescence measurement (FACS). (**C**,**D**) Three-gene activation together with one-gene repression. Transcription units and results from both RT-qPCR and FACS. In (B) and (D), denAsCas12a:ScRNA-5' provoked an unexpected, erroneous repression of *HED1* transcription. 'Ctrl' refers to the control circuits, where the pre-crRNA expression cassette is absent. Values on top of the bars refer to the activation/repression efficiency with respect to the corresponding control circuit.

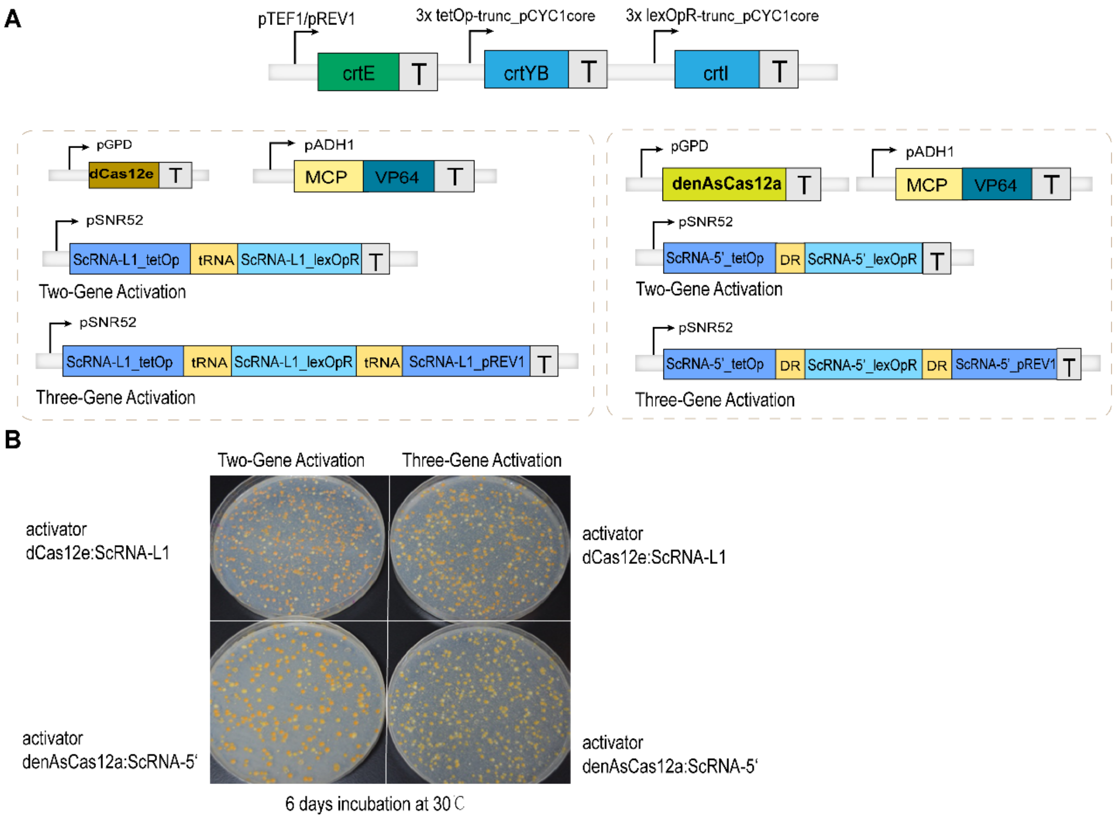

Figure S7. Synthetic β-carotene metabolic pathway. (**A**) Expression cassettes employed in β-carotene pathway activation. (**B**) Transformation plates. Different versions of the synthetic β-carotene pathways determine diverse tones of yeast-strain orange coloration. dCas12e:ScRNA-L1 always induces high β-carotene production, whereas denAsCas12a:ScRNA-5’ appears more effective when activating two genes.

2. Supplementary Tables With Statistical Data Analysis

Notation: In every table, each mean fluorescence intensity (indicated as FI) and standard deviation (SD) come from at least three independent experiments (unless otherwise specified). "Ratio vs Ctrl" is used as an abbreviation for "Ratio (full circuit/control circuit)", where a control circuit lacks the ScRNA/sgRNA/crRNA expression cassette (unless otherwise specified). As for statistical analysis, p-values are calculated via two-sided Welch's *t*-test (unless otherwise specified).

Table S1. The strength of synthetic and native yeast promoters used in this work (apart from pGAL1).

**Promoters (constitutive expression)**	**Mean FI (A.U.)**	**SD (A.U.)**	**Ratio versus pGPD (%)**
pCYC1core	1553.96	33.07	8.45
Truncated_pCYC1core	110.47	6.07	0.60
1xlexOpR-trunc_pCYC1core	264.98	12.20	1.44
3xlexOpR-trunc_pCYC1core	140.24	16.00	0.76
pGPD	18390.48	1210.51	100.00
pADH1	6943.21	580.39	37.75
pTEF2	8063.26	56.68	43.84
pTEF1	2741.91	238.05	14.91
pREV1	145.59	7.24	0.80
**Chemically regulated promoters**	**Mean FI (A.U.)**	**SD (A.U.)**	**Ratio OFF/ON (%)**
pMET25 (no methionine)	10155.77	345.19	-
pMET25 (10 mM methionine)	419.94	54.45	4.12
pGAL1 (2% galactose)	16963.46	465.92	-
pGAL1 (2% glucose)	43.74	36.25	0.26

Table S2. Data analysis, Figure S1C. dCas12e is fused to either VP64 or VPR. An sgRNA is expressed.

	**Mean FI (A.U.)**	**SD (A.U.)**	Ratio vs Ctrl	**p-value**	**Replicates**
dCas12e-VP64:sgRNA, circuit 1x					
Ctrl (no sgRNA)	95.13	13.42	-	-	3
bA	113.74	29.60	1.20	0.4815, (ns)	3
bS	157.02	14.99	1.65	0.0125, (*)	3
dCas12e-VP64:sgRNA, circuit 3x					
Ctrl (no sgRNA)	72.13	6.19	-	-	3
bA	68.43	16.01	0.95	0.7827, (ns)	3
bS	115.26	6.39	1.60	0.0024, (**)	3
dCas12e-VPR:sgRNA, circuit 1x					
Ctrl (no sgRNA)	136.49	2.98	-	-	3
bA	131.72	12.64	0.97	0.6509, (ns)	3
bS	339.97	18.11	2.49	0.0032, (**)	3
dCas12e-VPR:sgRNA, circuit 3x					
Ctrl (no sgRNA)	74.70	3.44	-	-	3
bA	158.16	10.19	2.12	0.0038, (**)	3
bS	263.23	2.89	3.52	<0.0001, (****)	3

Table S3. Data analysis, Figure 1D. dCas12e ScRNA has a single hairpin on loop 1. Circuit 1x.

	**Mean FI (A.U.)**	**SD (A.U.)**	**Ratio vs Ctrl**	**p-value**	**Replicates**
Ctrl (MCP)	79.31	1.42	-	-	4
MCP	290.65	13.37	3.66	<0.0001	4
Ctrl (PCP)	75.97	79.52	-	-	3
PCP	252.65	10.11	3.28	<0.0001	4
Ctrl (QCP)	92.55	5.16	-	-	4
QCP	200.63	7.12	2.17	<0.0001	4
Ctrl (Com)	154.21	11.45	-	-	3
Com	243.39	13.78	1.58	0.0024	3

Table S4. Data analysis, Figure 1E. dCas12e ScRNA has a single hairpin on loop 1. Circuit 3x. All 16 possible interactions between RNA hairpins and coat proteins are tested.

	Mean FI (A.U.)	SD (A.U.)	Ratio vs Ctrl	p-value	Replicates
**MCP**					
Ctrl (no ScRNA)	46.91	3.89	-	-	4
MS2	286.04	9.60	6.10	0.0003	3
PP7	25.94	3.56	0.55	0.0022	3
Qβ	34.15	5.30	0.73	0.0174	4
Com	30.52	2.75	0.65	0.0027	3
**PCP**					
Ctrl (no ScRNA)	48.15	6.61	-	-	3
MS2	52.58	4.87	1.09	0.4690	4
PP7	274.37	24.64	5.70	0.0010	4
Qβ	30.95	3.70	0.64	0.0480	4
com	36.66	5.19	0.76	0.1298	3
**QCP**					
Ctrl (no ScRNA)	53.23	9.83	-	-	4
MS2	48.67	5.79	0.91	0.5208	4
PP7	51.56	7.18	0.97	0.8213	4
Qβ	237.82	23.98	4.47	0.0003	4
com	52.50	11.83	0.99	0.7128	3
**Com**					
Ctrl (no ScRNA)	84.89	8.71	-	-	3
MS2	22.37	6.32	0.26	0.0018	3
PP7	37.41	8.18	0.44	0.0050	3
Qβ	24.01	2.82	0.28	0.0069	4
com	226.04	28.97	2.66	0.0143	3

Table S5. Data analysis, Figure 1F. dCas12e ScRNA has a double hairpin on loop 1. Circuits 1x and 3x.

	Mean FI (A.U.)	SD (A.U.)	Ratio vs Ctrl	p-value	Replicates
**MCP, circuit 1x**					
Ctrl (no ScRNA)	79.31	1.42	-	-	4
2xMS2	321.11	25.43	4.05	0.0005	4
**MCP, circuit 3x**					
Ctrl (no ScRNA)	46.91	3.89	-	-	4
2xMS2	610.89	37.14	13.02	<0.0001	5
**PCP, circuit 1x**					
Ctrl (no ScRNA)	75.97	79.52	-	-	3
2xPP7	449.62	2.78	5.83	<0.0001	3
**PCP, circuit 3x**					
Ctrl (no ScRNA)	48.15	6.61	-	-	3
2xPP7	339.62	16.72	7.05	0.0004	3

Table S6. Data analysis, Figure 2B. dCas12e ScRNA has a double hairpin (2xMS2) on one or two positions. Circuit 1x.

	Mean FI (A.U.)	SD (A.U.)	Ratio vs Ctrl	p-value	Replicates
Ctrl (no ScRNA)	79.31	1.42	-	-	4
Sc1	102.09	4.80	1.29	0.0170	3
Sc2	289.62	12.25	3.65	0.0016	3
Sc3	430.07	34.00	5.42	0.0046	3
Sc4	306.52	32.82	3.86	0.0102	3

Table S7. Data analysis, Figure 2C. dCas12e ScRNA has a double hairpin on one or two positions (2xMS2, Sc1-Sc4; 2xMS2 and 2xPP7, Sc5). Circuits 3x.

	Mean FI (A.U.)	SD (A.U.)	Ratio vs Ctrl	p-value	Replicates
Ctrl (no ScRNA, MCP is expressed)	46.91	3.89	-	-	4
Sc1	89.84	8.66	1.92	0.0117	3
Sc2	328.75	8.10	7.01	<0.0001	4
Sc3	319.86	15.89	6.82	0.0012	3
Sc4	338.76	19.39	7.22	0.0017	3
Ctrl (no ScRNA, MCP and PCP are expressed)	38.18	6.20	-	-	4
Sc5	341.92	26.95	8.96	<0.0001	5

Table S8. Data analysis, Figure 2D. dCas12e is fused to the VPR AD (dCas12e-VPR). The ScRNA has a double hairpin on loop 1. Circuit 3x.

	Mean FI (A.U.)	SD (A.U.)	Ratio vs Ctrl	p-value	Replicates
Ctrl (no ScRNA)	63.05	8.83	-	-	3
ScRNA-L1	348.78	51.76	5.53	0.0140	3

Table S9. Data analysis, Figure 2E. MCP is fused to two copies of the VP64 AD (MCP-2xVP64). dCas12e ScRNA has a double hairpin on loop 1. Circuit 1x and 3x.

	Mean FI (A.U.)	SD (A.U.)	Ratio vs Ctrl	p-value	Replicates
**Circuit 1x**					
Ctrl (no ScRNA)	71.99	4.42	-	-	3
ScRNA-L1	132.02	4.45	1.83	0.0002	3
**Circuit 3x**					
Ctrl (no ScRNA)	35.54	7.46	-	-	3
ScRNA-L1	122.24	14.70	3.44	0.0052	3

Table S10. Data analysis, Figure 3B. denAsCas12a ScRNA contains a double hairpin (2xMS2) at the 3' end (ScRNA-3'). Circuit 3x.

	Mean FI (A.U.)	SD (A.U.)	Ratio vs Ctrl	p-value	Replicates
Ctrl (no ScRNA-3')	35.61	4.05	-	-	5
ScRNA-3'	61.17	4.88	1.72	0.0052	3

Table S11. Data analysis, Figure 3C. denAsCas12a ScRNA contains a double hairpin (2xMS2) at the 5'-end (ScRNA-5'), Circuit 3x. Four mutated DR are employed (mDR1-4). The 2xMS2 is separated from the mDR by a linker of variable length (from 2 to 32 bp).

	Mean FI (A.U.)	SD (A.U.)	Ratio vs Ctrl	p-value	Replicates
Ctrl (no ScRNA-5')	35.61	4.05	-	-	5
**linker_16 (fixed)**					
mDR1	179.19	12.47	5.03	<0.0001	5
mDR2	139.02	9.68	3.90	<0.0001	4
mDR3	40.24	5.67	1.13	0.3772	3
mDR4	39.13	5.07	1.10	0.4499	3
**mDR1 (fixed)**					
linker_2	199.51	9.55	5.60	<0.0001	4
linker_5	224.55	7.96	6.31	<0.0001	4
linker_9	224.81	7.35	6.31	<0.0001	4
linker_12	180.12	2.59	5.06	<0.0001	3
linker_32	135.54	12.75	3.81	0.0060	3

Table S12. Data analysis, Figure S3A. denAsCas12a is fused to VP64. A crRNA is expressed. Circuit 3x.

	Mean FI (A.U.)	SD (A.U.)	Ratio vs Ctrl	p-value	Replicates
**denAsCas12a-VP64**					
- (Ctrl, no crRNA)	64.01	6.79	-	-	3
+ (with crRNA)	150.22	6.42	2.35	0.0002	3

Table S13. Data analysis, Figure S3B. MCP is fused to the P65-HSP1 AD and bind either ScRNA-L1 or ScRNA-5'.

	Mean FI (A.U.)	SD (A.U.)	Ratio vs Ctrl	p-value	Replicates
**dCa12e**					
- (Ctrl, no ScRNA-L1)	55.52	5.09	-	-	3
+ (ScRNA-L1)	20.35	0.40	0.37	0.0089	3
**denAsCas12a**					
- (Ctrl, no ScRNA-5')	37.85	6.96	-	-	3
+ (ScRNA-5')	16.84	4.44	0.44	0.0299	3

Table S14. Data analysis, Figure 4A. Each p-value was calculated via two-sided Welch's *t*-test by comparing the FI at a certain concentration of GA3 with that in the absence of GA3.

GA3 concentration (μM)	Mean FI (A.U.)	SD (A.U.)	Ratio vs Ctrl	p-value	Replicates
**YES gate sensing GA3, dCas12e:ScRNA-L1, MCP-GAI, GID1-VP64, circuit 1x**					
0	138.84	8.63	-	-	3
10	145.45	8.99	1.05	0.3739	4
15	154.79	4.69	1.11	0.0650	3
20	162.19	7.26	1.17	0.0242	3
25	228.25	25.60	1.64	0.0179	3
50	250.90	24.23	1.81	0.0088	3
75	268.30	7.17	1.93	<0.0001	3
100	269.38	23.96	1.94	0.0059	3
125	279.77	13.76	2.02	0.0003	3
150	298.89	24.16	2.15	0.0037	3
200	302.14	17.45	2.18	0.0008	3
300	320.64	29.74	2.31	0.0055	3
400	354.10	10.56	2.55	<0.0001	3
**GA3 concentration (μM)**	**Mean FI (A.U.)**	**SD (A.U.)**	**Ratio vs Ctrl**	**p-value**	**Replicates**
**YES gate sensing GA3, dCas12e:ScRNA-L1, MCP-GAI, GID1-VP64, circuit 3x**					
0	156.08	6.60	-	-	3
25	261.30	18.43	1.67	0.0053	3
50	326.38	29.73	2.09	0.0009	4
75	363.34	20.49	2.33	0.0015	3
100	369.93	20.49	2.37	0.0014	3
125	383.55	12.41	2.46	<0.0001	3
150	393.12	24.85	2.52	0.0022	3
200	405.84	20.51	2.60	0.0010	3
300	418.22	13.71	2.68	<0.0001	4
400	425.82	23.23	2.73	0.0013	3

Table S15. Data analysis, Figure S4B. Each p-value was calculated via two-sided Welch's *t*-test by comparing the FI at a certain concentration of GA3 with that in the absence of GA3.

**GA3 concentration (μM)**	**Mean FI (A.U.)**	**SD (A.U.)**	**Ratio vs Ctrl**	**p-value**	**Replicates**
**Control circuit for YES gate sensing GA3. ScRNA-L1 is not expressed (circuit 1x)**					
0	118.71	8.17	-	-	3
20	98.76	6.42	0.83	0.0564	3
50	93.77	6.54	0.79	0.0301	3
100	94.51	5.42	0.80	0.0314	3
150	88.75	2.80	0.75	0.0253	3
300	86.18	3.36	0.73	0.0185	3
**GA3 concentration (μM)**	**Mean FI (A.U.)**	**SD (A.U.)**	**Ratio vs Ctrl**	**p-value**	**Replicates**
**Control circuit for YES gate sensing GA3. ScRNA-L1 is not expressed (circuit 3x)**					
0	85.48	5.50	-	-	3
20	54.52	6.41	0.64	0.0070	3
50	56.56	2.76	0.66	0.0073	3
100	53.04	3.78	0.62	0.0036	3
150	48.03	2.21	0.56	0.0049	3
300	48.72	1.84	0.57	0.0063	3

Table S16. Data analysis, Figure 4B. Each p-value was calculated via two-sided Welch's *t*-test by comparing the FI at a certain concentration of ABA with that in the absence of ABA.

ABA concentration (μM)	Mean FI (A.U.)	SD (A.U.)	Ratio vs Ctrl	p-value	Replicates
**YES gate sensing ABA. dCas12e:ScRNA-L1, MCP-PYL1, ABI-VP64, circuit 1x**					
0	82.54	3.33	-	-	4
0.25	149.25	4.86	1.81	0.0003	3
0.5	173.01	7.30	2.10	0.0012	3
1	206.91	2.68	2.51	<0.0001	4
5	238.07	6.48	2.88	0.0002	3
10	214.96	5.36	2.60	<0.0001	3
15	210.00	10.04	2.54	0.0018	3
25	212.90	14.86	2.58	<0.0001	4
50	205.44	23.98	2.49	0.0175	3
75	204.24	10.30	2.47	0.0021	3
100	208.56	11.18	2.53	0.0001	4
125	205.26	11.48	2.49	0.0029	3
150	201.47	7.52	2.44	0.0007	3
200	212.75	3.67	2.58	<0.0001	3
**ABA concentration (μM)**	**Mean FI (A.U.)**	**SD (A.U.)**	**Ratio vs Ctrl**	**p-value**	**Replicates**
**YES gate sensing ABA. dCas12e:ScRNA-L1, MCP-PYL1, ABI-VP64, circuit 3x**					
0	33.55	0.95	-	-	3
0.25	136.84	0.93	4.08	<0.0001	3
0.5	172.78	7.33	5.15	<0.0001	4
1	227.19	6.29	6.77	0.0004	3
5	275.31	3.94	8.21	<0.0001	3
10	284.53	11.03	8.48	0.0009	3
15	277.46	14.61	8.27	0.0017	3
25	251.81	5.55	7.50	<0.0001	4
50	246.57	5.33	7.35	<0.0001	4
75	239.08	9.86	7.13	0.0010	3
100	247.51	5.39	7.38	<0.0001	4
125	242.41	10.46	7.22	0.0012	3
150	245.20	6.32	7.31	0.0003	3
200	243.29	9.05	7.25	0.0008	3

Table S17. Data analysis, Figure S4C. Each p-value was calculated via two-sided Welch's *t*-test by comparing the FI at a certain concentration of ABA with that in the absence of ABA.

ABA concentration (μM)	Mean FI (A.U.)	SD (A.U.)	Ratio vs Ctrl	p-value	Replicates
YES gate sensing ABA, dCas12e:ScRNA-L1, MCP-ABI, PYL1-VP64, circuit 3x – NOT WORKING					
0	10.53	1.99	-		3
0.25	11.06	1.52	1.05	0.7806	3
5	8.68	3.06	0.82	0.5196	3
15	7.89	1.32	0.75	0.2052	3
25	8.84	3.19	0.84	0.5662	3
100	6.50	3.52	0.62	0.2494	3
200	3.61	2.48	0.34	0.0394	3
**ABA concentration (μM)**	**Mean FI (A.U.)**	**SD (A.U.)**	**Ratio vs Ctrl**	**p-value**	**Replicates**
Control circuit for YES gate sensing ABA. ScRNA-L1 is not expressed (circuit 3x)					
0	40.54	2.80	-	-	3
0.25	36.99	6.43	0.91	0.5312	3
5	37.54	7.99	0.93	0.6584	3
25	33.42	6.66	0.82	0.2679	3
100	27.15	8.72	0.67	0.1525	3

Table S18. Data analysis, Figure S4D. Each p-value was calculated via two-sided Welch's *t*-test by comparing the FI at a certain concentration of ABA with that in the absence of ABA.

ABA concentration (μM)	Mean FI (A.U.)	SD (A.U.)	Ratio vs Ctrl	p-value	Replicates
Control circuit for YES gate sensing ABA. ScRNA-L1 is not expressed (circuit 1x)					
0	94.22	0.78	-	-	3
0.25	103.42	5.88	1.10	0.1553	3
50	108.14	3.71	1.15	0.0056	4
100	93.37	3.65	0.99	0.7758	3
150	97.00	5.05	1.03	0.5190	3
200	86.28	12.72	0.92	0.3591	4
**ABA concentration (μM)**	**Mean FI (A.U.)**	**SD (A.U.)**	**Ratio vs Ctrl**	**p-value**	**Replicates**
Control circuit for YES gate sensing ABA. ScRNA-L1 is not expressed (circuit 3x)					
0	58.05	2.50	-	-	3
0.25	58.94	8.17	1.02	0.8937	3
50	52.34	10.81	0.90	0.5358	3
100	62.15	2.29	1.07	0.1620	3
150	61.41	4.03	1.06	0.3825	3
200	44.74	7.27	0.77	0.1103	3

Table S19. Data analysis, Figure 4C. Each p-value was calculated via two-sided Welch's *t*-test by comparing the FI at a certain concentration of ABA and GA3 with that in the absence of both ABA and GA3.

**ABA and GA3 concentrations**	**Mean FI (A.U.)**	**SD (A.U.)**	**Ratio vs Ctrl**	**p-value**	**Replicates**
**AND gate sensing ABA and GA3. dCas12e:ScRNA-L1, MCP-PYL1, ABI-GAI, GID1-VP64, circuit 3x**					
0 µM ABA-0 µM GA3	28.96	2.67	-	-	3
5 µM ABA-0 µM GA3	79.63	1.74	2.75	<0.0001	3
0 µM ABA-100 µM GA3	34.95	0.47	1.21	0.0824	3
5 µM ABA-100 µM GA3	194.18	4.89	6.71	<0.0001	4
**Control circuit for AND gate sensing ABA and GA3. ScRNA-L1 is not expressed, circuit 3x**					
0 µM ABA-0 µM GA3	36.99	2.60	-	-	3
5 µM ABA-0 µM GA3	27.97	3.72	0.76	0.0548	3
0 µM ABA-100 µM GA3	34.02	5.18	0.92	0.5230	3
5 µM ABA-100 µM GA3	37.47	2.19	1.01	0.8517	3

Table S20. Data analysis, Figure 5A.

	**Mean FI (A.U.)**	**SD (A.U.)**	**Ratio vs Ctrl**	**p-value**	**Replicates**
**denAsCas12a:ScRNA-5', activation only, yEGFP**					
Ctrl (no ScRNA-5')	95.64	0.93	-	-	3
2TU	408.48	13.96	4.27	0.0009	3
PC	520.68	11.39	5.44	0.0003	3
**denAsCas12a:ScRNA-5', activation only, yEBFP**					
Ctrl (no ScRNA-5')	7.26	0.39	-	-	3
2TU	32.29	4.63	6.94	0.0006	3
PC	50.43	1.91	4.44	0.0161	3

Table S21. Data analysis, Figure 5B.

	Mean FI (A.U.)	SD (A.U.)	Ratio vs Ctrl	p-value	Replicates
**dCas12e:ScRNA-L1, activation, yEGFP**					
Ctrl (no ScRNA-L1)	164.46	2.93	-	-	3
2TU	949.37	26.14	5.77	0.0005	3
PC	745.41	23.95	4.53	<0.0001	4
**dCas12e:ScRNA-L1, activation, yEBFP**					
Ctrl (no ScRNA-L1)	5.81	0.83	-	-	3
2TU	27.14	6.68	4.67	0.0437	3
PC	30.28	4.11	5.21	0.0113	3

Table S22. Data analysis, Figure 5C.

	Mean FI (A.U.)	SD (A.U.)	Ratio vs Ctrl	p-value	Replicates
**denAsCas12a alone, activation and repression, yEGFP**					
Ctrl (no ScRNA-5’)	320.16	10.30	-	-	4
PC	1700.81	16.39	5.43	<0.0001	4
**denAsCas12a alone, activation and repression, yEBFP**					
Ctrl (no ScRNA-5’)	6613.66	79.59	-	-	4
PC	1427.91	42.07	0.22	<0.0001	4

Table S23. Data analysis, Figure 5D.

	Mean FI (A.U.)	SD (A.U.)	Ratio vs Ctrl	p-value	Replicates
**dCas12e and denAsCas12a, activation and repression, yEGFP**					
Ctrl (no crRNA, no ScRNA-L1)	226.74	11.29	-	-	4
PC	1139.20	82.40	5.02	0.0003	4
**dCas12e and denAsCas12a, activation and repression, yEBFP**					
Ctrl (no crRNA, no ScRNA-L1)	3305.97	123.25	-	-	4
PC	756.13	27.17	0.23	<0.0001	4

Table S24. Data analysis, Figure 5E.

	Relative mRNA level	SD	Ratio vs Ctrl	p-value	Replicates
**denAsCas12a:ScRNA-5', 2TU**					
Ctrl (HED1)	1.01	0.14	-	-	3
HED1	3.52	0.38	3.48	0.0035	3
Ctrl (yEGFP)	1.01	0.10	-	-	3
yEGFP	9.95	0.53	9.89	0.0008	3
	**Relative mRNA level**	**SD**	**Ratio vs Ctrl**	**p-value**	**Replicates**
**denAsCas12a:ScRNA-5', PC**					
Ctrl (HED1)	1.01	0.14	-	-	3
HED1	2.07	0.26	2.06	0.0078	3
Ctrl (yEGFP)	1.01	0.10	-	-	3
yEGFP	7.90	0.31	7.84	0.0002	3
	**Relative mRNA level**	**SD**	**Ratio vs Ctrl**	**p-value**	**Replicates**
**dCas12e:ScRNA-L1, PC**					
Ctrl (GAL7)	1.00	0.10	-	-	3
GAL7	2.76	0.34	2.76	0.0080	3
Ctrl (yEGFP)	1.03	0.08	-	-	3
yEGFP	4.89	0.27	4.74	0.0007	3

Table S25. Data analysis, Figure S5E-F.

	Relative mRNA level	SD	Ratio vs Ctrl	p-value	Replicates
**denAsCas12a:ScRNA-5', PC**					
Ctrl (GAL7)	1.02	0.21	-	-	3
GAL7	1.69	0.20	1.66	0.0162	3
Ctrl (yEGFP)	1.01	0.10	-	-	3
yEGFP	14.39	0.39	14.29	0.0001	3
	**Relative mRNA level**	**SD**	**Ratio vs Ctrl**	**p-value**	**Replicates**
**dCas12e:ScRNA-L1, 2TU**					
Ctrl (GAL7)	1.00	0.06	-	-	3
GAL7	1.88	0.03	1.87	0.0002	3
Ctrl (yEGFP)	1.00	0.07	-	-	3
yEGFP	8.20	0.37	8.18	0.0006	3
	**Relative mRNA level**	**SD**	**Ratio vs Ctrl**	**p-value**	**Replicates**
**dCas12e:ScRNA-L1, 2TU**					
Ctrl (CYC1)	1.00	0.08	-	-	3
CYC1	1.47	0.11	1.47	0.0052	3
Ctrl (yEGFP)	1.00	0.07	-	-	3
yEGFP	7.92	0.50	7.91	0.0015	3

Table S26. Data analysis, Figure 5F.

	Mean FI (A.U.)	SD (A.U.)	Ratio vs Ctrl	p-value	Replicates
**Converter, yEGFP**					
Ctrl (no ScRNA-5', no Cre)	402.66	10.67	-	-	4
- Cre	2100.72	177.04	5.22	0.0005	4
+ Cre	216.97	7.31	0.54	<0.0001	4
**Converter, yEGFP**					
- Cre	2100.72	177.04	-	-	4
+Cre	216.97	7.31	-9.68	0.0003	4
**Converter, yEBFP**					
Ctrl (no crRNA-5', no Cre)	7972.16	467.59	-	-	3
- Cre	8944.23	209.67	0.89	0.0815	3
+ Cre	3430.31	114.61	-2.32	0.0035	3
**Converter, yEBFP**					
- Cre	8944.23	209.67	-	-	3
+ Cre	3430.31	114.61	-2.61	<0.0001	3

Table S27. Data analysis, Figure S5G.

	Mean FI (A.U.)	SD (A.U.)	Ratio vs Ctrl	p-value	Replicates
**Converter-Met, yEGFP**					
Ctrl (no ScRNA-5', no Cre)	402.66	10.67	-	-	4
+ Met	1011.47	33.37	2.51	0.0008	3
- Met	552.13	21.33	1.37	0.0002	4
**Converter-Met, yEGFP**					
+ Met (ctrl)	1011.47	33.37	-	-	3
- Met	552.13	21.33	-1.83	0.0004	4
**Converter-Met, yEBFP**					
Ctrl (no crRNA-5', no Cre)	7972.16	467.59	-	-	3
+ Met	5070.71	185.36	-1.57	0.0063	3
- Met	3567.18	94.78	-2.23	0.0043	3
**Converter-Met, yEBFP**					
+ Met (ctrl)	5070.71	185.36	-	-	3
- Met	3567.18	94.78	-1.42	0.0021	3

Table S28. Data analysis. Figure 6A.

	Relative mRNA level	SD	Ratio vs Ctrl	p-value	Replicates
**dCas12e:ScRNA-L1, four-gene activation**					
Ctrl (GAL7)	1.00	0.06	-	-	3
GAL7	1.71	0.03	1.70	0.0004	3
Ctrl (yEGFP)	1.00	0.07	-	-	3
yEGFP	3.00	0.16	3.00	0.0005	3
Ctrl (CYC1)	1.00	0.04	-	-	3
CYC1	1.39	0.07	1.39	0.0029	3
Ctrl (yEBFP)	1.00	0.07	-	-	3
yEBFP	2.91	0.37	2.90	0.0102	3

Table S29. Data analysis. Figure 6B.

	Relative mRNA level	SD	Ratio vs Ctrl	p-value	Replicates
**dCas12e:ScRNA-L1 (three-gene activation) and denAsCas12a:crRNA (one gene repression)**					
Ctrl (GAL7)	1.00	0.01	-	-	3
GAL7	1.52	0.11	1.51	0.0140	3
Ctrl (yEGFP)	1.00	0.08	-	-	3
yEGFP	3.91	0.39	3.90	0.0045	3
Ctrl (CYC1)	1.00	0.05	-	-	3
CYC1	1.50	0.08	1.50	0.0017	3
Ctrl (yEBFP)	1.00	0.04	-	-	3
yEBFP	0.22	0.02	0.22	<0.0001	3

Table S30. Data analysis. Figure S6B.

	Relative mRNA level	SD	Ratio vs Ctrl	p-value	Replicates
**denAsCas12a:ScRNA-5’, four-gene activation**					
Ctrl (HED1)	1.00	0.06	-	-	3
HED1	0.29	0.01	0.29	0.0019	3
Ctrl (yEGFP)	1.00	0.10	-	-	3
yEGFP	7.59	0.09	7.57	<0.0001	3
Ctrl (yEBFP)	1.01	0.10	-	-	3
yEBFP	7.15	0.53	7.10	0.0018	3
Ctrl (GAL7)	1.00	0.02	-	-	3
GAL7	1.64	0.08	1.64	0.0034	3

Table S31. Data analysis. Figure S6D.

	Relative mRNA level	SD	Ratio vs Ctrl	p-value	Replicates
**denAsCas12a:ScRNA/crRNA activation and repression**					
Ctrl (HED1)	1.00	0.01	-	-	3
HED1	0.31	0.03	0.31	0.0002	3
Ctrl (yEGFP)	1.00	0.05	-	-	3
yEGFP	7.22	0.57	7.19	0.0026	3
Ctrl (yEBFP)	1.01	0.12	-	-	3
yEBFP	0.79	0.04	0.79	0.0742	3
Ctrl (GAL7)	1.00	0.03	-	-	3
GAL7	1.53	0.05	1.53	0.0003	3

3. ScRNA

1. ScRNA construction—dCas12e

dCas12e sgRNA sequence (5'-3'):
GGCGCGTTTATTCCATTACTTTGGAGCCAGTCCCAGCGACTATGTCGTATGGACGAAGCGCT TATTTATCGGAGAGAAACCGATAAGTAAAACGCATCAAAG+ 'sp' sequences
In black: tracrRNA; blue: loop 2; red: loop 1; orange: DR.

The four viral RNA hairpin sequences (5'-3'):
1xMS2: gcACATGAGGATCACCCATGTgc
1xPP7 (PP7-LSm) (6): caTACGGAGTTTATATGGAAACCCGTAtg
1xQβ (Qβ-U(+6)C) (6): gcATGCATGTCCAAGACAGCATgc
1xcom: CTGAATGCCTGCGAGCATC
2xMS2 (similar sequence to 2x(wt+f6)MS2) (7):
agcACATGAGGATCACCCATGTgcgactcCCACAGTCACTGGGgagtctt
2xPP7 (7):
gggagcTACGGAGTTTATATGGAAACCCGTAgcctgctgcgTACGGAGTTTATATGGAAACCCGT Acgcag
Cagttccc

Spacers ('sp') sequences for dCas12e:ScRNA binding yEGFP (5'-3'):
bA (binding lexOpR on the anti-sense DNA strand): ATAACTGTATATACACCCAG
bS (binding lexOpR on the sense DNA strand): CCTGGGTGTATATACAGTTA

During the design of dCas12e ScRNA, an RNA hairpin was placed in one or two of the three regions selected to for crRNA engineering, i.e., the 5'-end, loop 1, and loop 2. The spacer was always 'bS'. We checked the ScRNA secondary structure with RNAfold (8) to ensure that the structure of tracrRNA and DR were not disrupted by the extra hairpins.

Note: in loop 1, RNA hairpins fully replaced the original DNA nucleotides; in loop 2, RNA hairpins were inserted between the fifth (A) and the sixth (T) base.

2. ScRNA construction—dSpCas9

sgRNA sequence of (d)Cas9 (5'-3'):
CATAACTGTATATACACCCAGTTTTAGAGCTAGA***n***AATAGCAAGTTAAAATAAGGCTAGTCC GTTATCAACTTGAAAAAGTGGCACCGAGTCGGTGC
Blue: spacer sequence; orange: Cas9 DR; red: loop; black: tracrRNA

Note: the construction of the ScRNAs for dSpCas9-based activators is similar to that followed for dCas12e. The ScRNA with 2xMS2 in the loop region were realized by placing 2xMS2 on the '***n***' region. '***n***', here, means 'nothing' (refers to the sgRNA) or '2xMS2 structure'. The ScRNA secondary structure was checked with RNAfold.

3. ScRNA construction—denAsCas12a

crRNA for denAsCas12a (5’-3’):

TAATTTCTACTCTTGTAGATCATAACTGTATATACACCCA

Orange: denAsCas12a DR; black: spacer binding the anti-sense lexOpR sequence (1). Bases highlighted in gray were subjected to mutations during mDRs construction.

The linker used when testing mDRs (5’-3’)

16bp linker: Ttccttccccttcccc

Linkers tested together with mDR1 (5’-3’)

2bp: tt

5bp: ttcct

9bp: ttccttctc

12bp: ttccttcccctt

32bp: ttccttccccttccccttccttccccttcccc

The construction steps are the same as in the construction of the dCas12e ScRNA. After designing an ScRNA for denAsCas12a, we checked its secondary structure with RNAfold.

4. RT-qPCR

Endogenous gene targets determination and primers used in RT-qPCR

Targets
pHED1: ACGGCTTTAATTAGCGTACG (for denAsCas12a)
pGAL7: CTTAACCCAAAAATAAGGGA (for denAsCas12a)
pGAL7: GATCACGGTCAACAGTTGTC (for dCas12e)
pCYC1: GGTAGGAAATTGATTACATC (for dCas12e)

Primers
HED1
Forward: AAGAGCTTGTGCACCGAAGT
Reverse: TGGCACGAAGTTGTTGTTTT

GAL7
Forward: GCCATTCCCATAGACGTTACA
Reverse: GCTTGTAAGCAGCCTCCTGT

CYC1
Forward: CAAGGCCGGTTCTGCTAAGA
Reverse: AGCTTGACCAGAGTGTCTGC

ACT1
Forward: CAGGTATTGCCGAAAGAA
Reverse: CCACATTTGTTGGAAGGTA

pHED1, pGAL7, and pCYC1 sequences, i.e., the endogenous targets for dCas12e- and denAsCas12a-based activators are taken from the NCBI database. The ScRNA binding sites were identified according to the location of the PAM and the TATA box. dCas12e: the PAM is 5’-TTCN and the target sequence was taken from the sense strand. denAsCas12a: the PAM is 5’-TTTV and the target sequence was taken from the anti-sense strand.

5. RNA-seq

In order to activate the expression of the two reporter proteins, yEGFP and yEBFP, we chose, as targets, the 20-nt-long bacterial sequences lexOpR and tetOp, respectively. We carried out a computational analysis, with the web-server Cas-OFFinder (9) (http://www.rgenome.net/cas-offinder/) to ensure that both bacterial operators are absent from the yeast genome (in conjunction with the PAM of both Cas12 proteins). The same analysis was repeated on the sequences of the endogenous promoters (pHED1, pGAL7, and pCYC1) where the dCas12:ScRNA activators bound. In this case, we wanted to verify their uniqueness in the *S. cerevisiae* genome. The results, presented in Table S32, point out that at least 4 mismatches are present between each target and its most similar genomic sequence.

Table S32. Computational OFF-target analysis. For each target, Cas-OFFinder finds sequences, downstream of the selected PAM, that differ for up to 9 mismatches. Perfect matches are present only for the endogenous genes. DNA and RNA Bulge Size were set to 0 (default value).

**Target name**	**Target sequence**	**Number of minimal mismatches**	**Recurrence in the genome**
dCas12e (TTCN-target)			
lexOpR	CCTGGGTGTATATACAGTTA	5	1
tetOp	TCTCTATCACTGATAGGGAG	4	1
pGAL7	GATCACGGTCAACAGTTGTC	0, 5	1, 1
pCYC1	GGTAGGAAATTGATTACATC	0, 4	1, 1
denAsCas12a (TTTV-target)			
lexOpR	CATAACTGTATATACACCCA	5	2
tetOp	CTCCCTATCAGTGATAGAGA	5	2
pHED1	ACGGCTTTAATTAGCGTACG	0, 5	1, 1
pGAL7	CTTAACCCAAAAATAAGGGA	0, 4	1, 1

RNA-seq analysis was carried out by (Sangon Biotech (Shanghai) Co., Ltd.). We selected two strains, byMM1827 and byMM1832, where both denAsCas12a:ScRNA-5' and dCas12e:ScRNA-L1 targeted 3xlexOpR-truncated_pCYC1core (in front of *yEGFP*) and the promoter of an endogenous gene (pHED1 and pGAL7, respectively). Transcript amount was compared to that of the corresponding "control" strains (byMM1777 and byMM1736), where the ScRNAs were not expressed. As shown in Figure S8, the R^2 values are very close to 1, which indicates that most of the yeast genes do not resent of the presence of dCas12:ScRNA activators. The *HIS3* gene, which is unfunctional in the control strains, is highly expressed in both strains hosting the complete circuits. The synthetically activated genes appear less expressed if compared to the RT-qPCR measurements (see Table S39). Rather strikingly, both byMM1827 and byMM1832

present a substantial number of genes that are either up- or even downregulated in the presence of dCas12:ScRNAs (their functions and possible cause of unbalanced expression are listed in Tables S40-S41). This is particularly evident when dCas12e:ScRNA-L1 is active in yeast strains.

Taken together, our computational analysis together with the RNA-seq results suggest that the expression and activity of (especially) dCas12e:ScRNA-L1 determine an overall stress condition for yeast cells. As a consequence, the transcription of multiple DNA sequences changes, also in a considerable way, which, however, does not preclude the normal cell functions and viability.

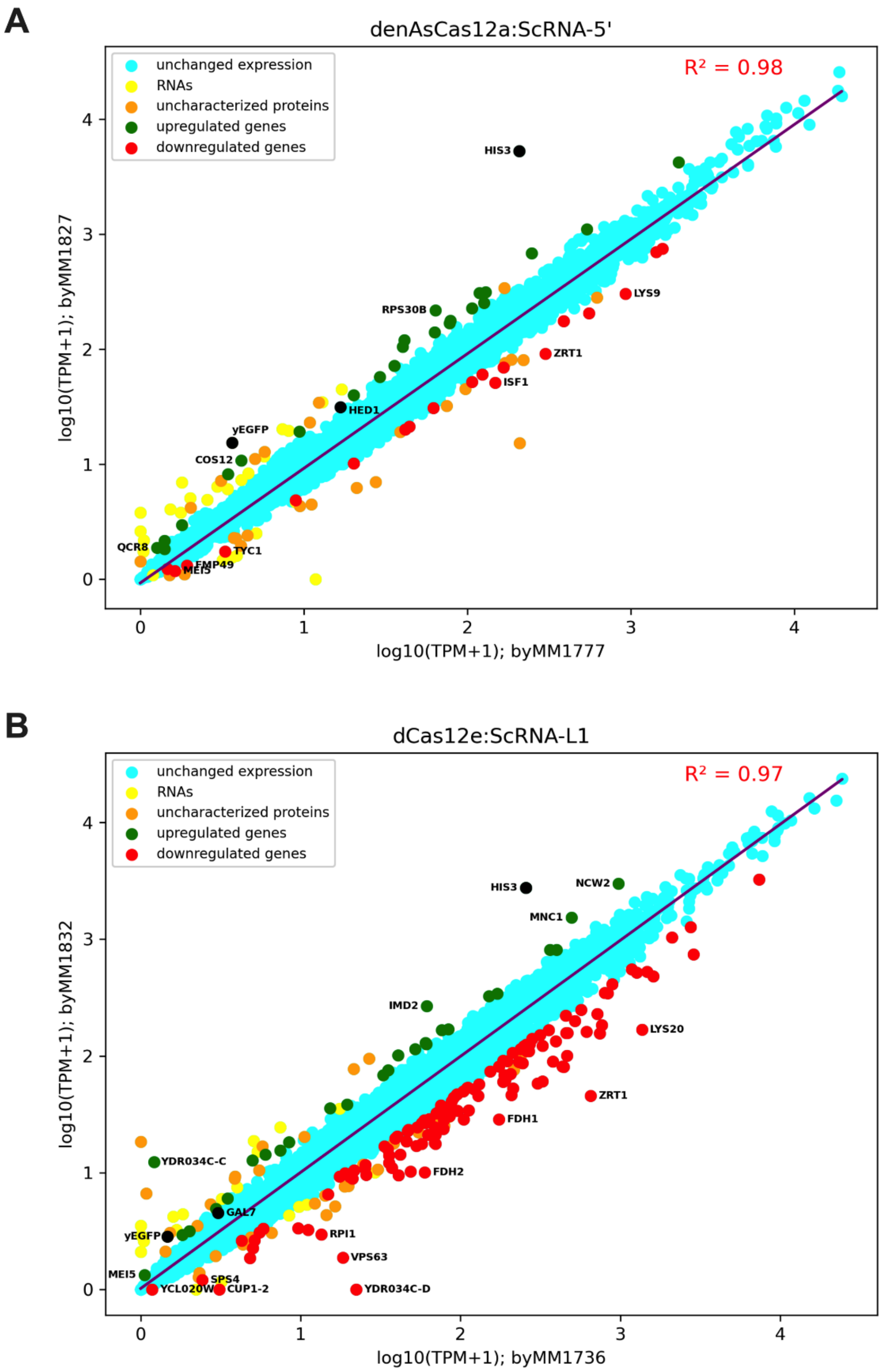

Figure S8. RNA-seq analyses. Effects on transcript abundance due to (**A**) denACas12a:ScRNA-5’ and (**B**) dCas12e:ScRNA-L1. Many over/under-expressed DNA segments are responsible for the production of RNA sequences (yellow dots) or uncharacterized proteins (orange dots). Black dots are the three genes (per strain) that are expected to be upregulated by our synthetic activators. Among the remaining genes, we labeled and described only those whose expression changed (up or down) in a remarkable way (see Tables S40-S41).

Table S33. Comparison between the transcript increase measured with RNA-seq and RT-qPCR.

	Gene	RNA-seq	RT-qPCR	Ratio
byMM1827 (denAsCas12a:ScRNA-5’)				
	yEGFP	5.43	9.89	1.82
	HED1	1.92	3.48	1.81
	**Gene**	**RNA-seq**	**RT-qPCR**	**Ratio**
byMM1832 (dCas12e:ScRNA-L1)				
	yEGFP	3.97	8.18	2.06
	GAL7	1.72	1.87	1.09

Table S34. Gene expression, byMM1827 vs byMM1777.

Gene ID	Gene Name	$\log_2$(fold change)	Notes (from SGD)
**Up-regulated genes—$\log_2$(fold change) > 1.59**			
YOR182C	RPS30B	1.79	Protein component of the small (40S) ribosomal subunit. Protein abundance **increases** in response to **DNA replication stress**.
YJL166W	QCR8	1.74	Subunit 8 of ubiquinol cytochrome-c reductase (Complex III). **Expression is regulated by Abf1p** (-0.18, **inv**) **and Cbf1p** (-0.13, **inv**).
YGL263W	COS12	1.63	Required to internalize plasma membrane proteins for degradation.
**Gene ID**	**Gene Name**	**$\log_2$(fold change)**	**Notes (from SGD)**
**Down-regulated genes—$\log_2$(fold change) < -1.50**			
YMR081C	ISF1	-1.56	Serine-rich, hydrophilic protein.
YER038W-A	FMP49	-1.57	Mitochondrial protein.
YNR050C	LYS9	-1.61	Saccharopine dehydrogenase, **Lysine requiring**.
YBR296C-A	TYC1	-1.62	Inhibitor of the Anaphase-Promoting Complex/Cyclosome (APC/C).
YGL255W	ZRT1	-1.73	**Transcription is induced under low-zinc conditions by Zap1p (inv).**
YPL121C	MEI5	-1.78	Meiosis-specific protein.

Table S35. Gene expression, byMM1832 vs byMM1736.

**Gene ID**	**Gene Name**	**log_2(fold change)**	**Notes (from SGD)**
Up-regulated genes—log_2(fold change) > 1.59			
YDR034C-C	YDR034C-C	5.71	Retrotransposon TYA Gag gene.
YPL121C	MEI5	2.55	Meiosis-specific protein. Forms heterodimer with Sae3p (inv).
YHR216W	IMD2	2.14	Inosine monophosphate dehydrogenase. **Expression is induced by mycophenolic acid** resulting in resistance to drugs.
YBR056W-A	MNC1	1.64	Putative membrane protein. **Upregulated by toxic concentrations of heavy metal ions (e.g., Mn^{2+}, Co^{2+}, Ni^{2+}, Cu^{2+}, and Zn^{2+}).**
YLR194C	NCW2	1.62	GPI-anchored protein involved in cell wall remodeling. **Expression is upregulated in response to cell wall stress.**
**Gene ID**	**Gene Name**	**log_2(fold change)**	**Notes (from SGD)**
Down-regulated genes—log_2(fold change) < -2.50			
YOR388C	FDH1	-2.65	Formate DeHydrogenase, may protect cells from exogenous formate.
YIL119C	RPI1	-2.67	Mediates fermentation stress tolerance by modulating cell wall integrity.
YPL275W	FDH2	-2.70	Formate DeHydrogenase, may protect cells from exogenous formate.
YOR313C	SPS4	-2.79	Protein whose **expression is induced during sporulation**.
YDL182W	LYS20	-3.03	Homocitrate synthase isozyme, functions in DNA repair. LYSine requiring
YGL255W	ZRT1	-3.87	Zinc-Regulated Transporter, **transcription is induced under low-zinc conditions by the Zap1p (inv)**.
YLR261C	VPS63	-4.30	Vacuolar Protein Sorting. Decreased level in enolase-deficient mutants.
YCL020W	YCL020W	-10.81	Retrotransposon TYA Gag gene.
YHR055C	CUP1-2	-14.36	Metallothionein; binds copper and mediates resistance to high concentrations of copper and cadmium.
YDR034C-D	YDR034C-D	-17.70	Retrotransposon TYA Gag.

Source: the Saccharomyces Genome Database (SGD)—https://www.yeastgenome.org

References

1. Yu, L. and Marchisio, M.A. (2021) Saccharomyces cerevisiae Synthetic Transcriptional Networks Harnessing dCas12a and Type V-A anti-CRISPR Proteins. *ACS Synth Biol*.
2. Liu, J.J., Orlova, N., Oakes, B.L., Ma, E., Spinner, H.B., Baney, K.L.M., Chuck, J., Tan, D., Knott, G.J., Harrington, L.B. *et al.* (2019) CasX enzymes comprise a distinct family of RNA-guided genome editors. *Nature*, **566**, 218-223.
3. Guo, M., Ren, K., Zhu, Y., Tang, Z., Wang, Y., Zhang, B. and Huang, Z. (2019) Structural insights into a high fidelity variant of SpCas9. *Cell Res*, **29**, 183-192.
4. Atasoy, D., Aponte, Y., Su, H.H. and Sternson, S.M. (2008) A FLEX switch targets Channelrhodopsin-2 to multiple cell types for imaging and long-range circuit mapping. *J Neurosci*, **28**, 7025-7030.
5. Yu, L. and Marchisio, M.A. (2023) CRISPR-associated type V proteins as a tool for controlling mRNA stability in S. cerevisiae synthetic gene circuits. *Nucleic acids research*.
6. Katz, N., Cohen, R., Solomon, O., Kaufmann, B., Atar, O., Yakhini, Z., Goldberg, S. and Amit, R. (2018) An in Vivo Binding Assay for RNA-Binding Proteins Based on Repression of a Reporter Gene. *ACS Synth Biol*, **7**, 2765-2774.
7. Zalatan, J.G., Lee, M.E., Almeida, R., Gilbert, L.A., Whitehead, E.H., La Russa, M., Tsai, J.C., Weissman, J.S., Dueber, J.E., Qi, L.S. *et al.* (2015) Engineering complex synthetic transcriptional programs with CRISPR RNA scaffolds. *Cell*, **160**, 339-350.
8. Denman, R.B. (1993) Using RNAFOLD to predict the activity of small catalytic RNAs. *BioTechniques*, **15**, 1090-1095.
9. Bae, S., Park, J. and Kim, J.S. (2014) Cas-OFFinder: a fast and versatile algorithm that searches for potential off-target sites of Cas9 RNA-guided endonucleases. *Bioinformatics (Oxford, England)*, **30**, 1473-1475.
